# High-Fat Diet Alters the Expression of Reference Genes in Male Mice

**DOI:** 10.3389/fnut.2020.589771

**Published:** 2020-11-24

**Authors:** Xiuqin Fan, Hongyang Yao, Xuanyi Liu, Qiaoyu Shi, Liang Lv, Ping Li, Rui Wang, Tiantian Tang, Kemin Qi

**Affiliations:** ^1^Laboratory of Nutrition and Development, Beijing Pediatric Research Institute, Beijing Children's Hospital, Capital Medical University, National Center for Children's Health, Beijing, China; ^2^Zeesan Biotech, Xiamen, China

**Keywords:** reference genes, qPCR, obesity, mice, adipose tissue, liver

## Abstract

Quantitative PCR (qPCR), the most accurate and sensitive technique for quantifying mRNA expression, and choice of appropriate reference genes for internal error controlling in qPCR are essential to understanding the molecular mechanisms that drive the obesity epidemic and its comorbidities. In this study, using the high-fat diet (HFD)-induced obese mouse model, we assessed the expression of 10 commonly used reference genes to validate gene-expression stability in adipose tissue, liver, and muscle across different time points (4, 8, 12, and 16 weeks after HFD feeding) during the process of obesity. The data were analyzed by the GeNorm, NormFinder, BestKeeper, and Delta-Ct method, and the results showed that the most stable reference genes were different for a specific organ or tissue in a specific time point; however, PPIA, RPLP0, and YWHAZ were the top three most stable reference genes in qPCR experiments on adipose, hepatic tissues, and muscles of mice in diet-induced obesity. In addition, the mostly used genes ACTB and GAPDH were more unstable in the fat and liver, the ACTB mRNA levels were increased in four adipose tissues, and the GAPDH mRNA levels were decreased in four adipose tissues and liver after HFD feeding. These results suggest that PPIA, RPLP0, or YWHAZ may be more appropriate to be used as reference gene than ACTB and GAPDH in the adipose tissue and liver of mice during the process of high-fat diet-induced obesity.

## Introduction

The rapidly increasing prevalence of obesity worldwide and its associated metabolic complications, such as non-alcoholic fatty liver, dyslipidemia, and type 2 diabetes, have become a threat for human health (1, 2). To investigate the underlying mechanisms, a variety of tools and techniques including metabolic, proteomic, transcriptomic, and novel DNA sequencing strategies have been employed, among which quantitative PCR (qPCR) and reverse transcription (RT)-qPCR are the most accurate and sensitive techniques for quantifying mRNA in biological samples and have become accessible to virtually all research labs (3–5). However, there remain a number of problems associated with qPCR use, including variability of sample preparation, extraction and storage, RNA isolation and purification, RT, poor choice of primers, and inappropriate reference targets (3–5). In 2009, the minimum information for the publication of quantitative real-time PCR experiments (MIQE) was published to provide the scientific community with a consistent workflow and key considerations to perform qPCR experiments (4). However, the MIQE standards have not been embraced more widely in practice.

Normalizing to a reference gene, whose expression has to be stable and independent of the experimental conditions, is a key step for internally controlling for error in qPCR (4–6). During the past decades, β-actin (ACTB), glyceraldehyde-3-phosphate dehydrogenase (GAPDH), 18S ribosomal RNA (18S), ribosomal protein large P0 (RPLP0), and TATA box-binding protein (TBP) have been used extensively as reference genes in physiological status and diseases including obesity (7–10). However, increasing evidence suggests that the expression of reference genes often varies considerably with differences in subjects, animal species, experimental models, disease conditions, tissue types, etc. (11, 12). Therefore, it is essential to validate potential reference genes to establish whether they are appropriate for a specific experimental purpose.

In recent years, several research groups have evaluated stability of reference genes for qPCR in human and mouse adipose tissue by different methods of mathematical algorithms (7, 13–17). However, consistent conclusions have not been reached owing to constant changes in fat accumulation of adipose tissue and associated cell size with the development of obesity, different analyzing methods used, etc.

Therefore, in this study, after reviewing the literature, a total of 10 commonly used reference genes involved in different biological functions, including ACTB, GAPDH, hypoxanthine phosphoribosyl transferase 1 (HPRT), 18S, RPLP0, beta-2-microglobulin (B2M), TBP, peptidylprolyl isomerase A (PPIA), ubiquitin C (UBC), and tyrosine 3-monooxygenase/tryptophan 5-monooxygenase activation protein, and zeta polypeptide (YWHAZ), were determined to analyze gene-expression stability in tissues (adipose tissue, liver, and muscle) associated with energy and fat metabolism across different time points during the development of obesity in mice, using the NormFinder (18), GeNorm (11), BestKeeper (19), and Delta-Ct method (20).

## Methods and Materials

### Animal Procedures

Three- to four-week-old male C57BL/6J mice were purchased from the SPF Laboratory Animal Technology Co., Ltd (Beijing), and were housed at the animal facilities in the National Institute of Occupational Health and Poison Control, China CDC, under a 12-h (h) light 12-h dark cycle with cycles of air ventilation and constant temperature (23°C), with free access to water and food. After 1 week of recovery from transportation, the mice were randomly divided into two groups (*n* = 32 in each group) and fed with a high-fat diet (HFD) (34.9% fat by wt., 60% kcal) (No. H10060) and a normal-fat diet (NFD) (4.3% fat by wt., 10% kcal) (No. H10010) (Beijing Huafukang Bioscience Co. Inc., Beijing, China) based on formulas of the high-fat diets for DIO mice (D12492) and the paired control diet (D12450B) (Research Diets, New Brunswick, NJ, USA). The fat in both of the diets was from soybean oil and lard oil, and the diet formula was shown in Supplementary Table 1. The diets were sterilized with γ-irradiation 25 kGy and stored at −20°C until use.

Mouse body weight was measured weekly, and food consumption was detected at 4, 8, 12, and 16 weeks after feeding with 7 consecutive days of records. At 4, 8, 12, and 16 weeks after feeding respectively, the 12-h fasted mice (*n* = 8 in each group) in a fasted state were euthanized by intraperitoneal injection of an overdose of Avertin (500 mg kg^−1^ of 2,2,2-tribromoethanol, T-4840-2, Sigma-Aldrich Chemie GmbH, Steinheim, Germany) to minimize suffering. After euthanization, the epididymal, perirenal, subcutaneous inguinal fat, subscapular brown adipose tissue, liver, and femoral muscle were immediately dissected free of the surrounding tissue, removed, wrapped in aluminum foil, and frozen in liquid N2 and then were transferred to −80°C until use.

### RT-qPCR for Candidate Reference Genes

Total RNA in tissues was prepared using the TRIzol Reagent kit (Invitrogen, Carlsbad, CA, USA). Briefly, 80 mg of epididymal, perirenal, or subcutaneous inguinal fat tissues and 20 mg of subscapular brown adipose tissue, liver, or femoral muscle were homogenized in 1 mL of TRIzol reagent. After centrifugation, RNA was extracted with chloroform and precipitated with isopropyl alcohol, then resuspended in 30-100 μL of DEPC-treated water, and finally its concentration and purity in each sample were determined by a DS-11 Spectrophotometer (DeNovix) (Supplementary Table 2). One microgram of extracted RNA in each sample was used for reverse-transcribed cDNA First-strand (cDNA) synthesis using the All-in-One First-Strand cDNA Synthesis SuperMix for qPCR (One-Step gDNA Removal) (TransGen Biotech, Beijing, China) according to the procedures provided by the manufacturer. The mRNA expression of targeted genes including ACTB, GAPDH, 18S, HPRT, RPLP0, B2M, TBP, PPIA, UBC, and YWHAZ was measured by real-time qPCR with a CFX96 Touch™ Real-Time PCR Detection System (Bio-Rad) using Top Green qPCR SuperMix (Trans Gen). The oligonucleotide primers for these target genes were from the PrimerBank (https://pga.mgh.harvard.edu/primerbank/), and the published papers (7, 21, 22) were tested for specificity using Primer-BLAST (https://www.ncbi.nlm.nih.gov/tools/primer-blast/index.cgi?LINK_LOC=BlastHome), showing all to be validated with over 90% efficiency in amplification (Table 1). Each reaction was performed in the final volume of 20 μL including 1 μL of cDNA and 200 nM of each primer, with the thermocycle program consisting of an initial hot start cycle at 95°C for 30 s, followed by 40 cycles at 95°C for 5 s, 60°C for 15 s, and 72°C for 10 s. The specificity of the amplification was analyzed by agarose gel electrophoresis (Supplementary Figure 1) and melting curves (Supplementary Figure 2).

**Table 1:** Detail of primers used for each of the 10 evaluated reference genes.

**Gene symbol**	**Gene name**	**Gene function**	**Primer sequence (5^**′**^-3^**′**^)**	**Amplicon length (bp)**	**Efficiency (%)**
ACTB	Actin beta	Cytoskeletal structural protein	GTGACGTTGACATCCGTAAAGA GCCGGACTCATCGTACTCC	245	97.0
GAPDH	Glyceraldehyde-3-phosphate dehydrogenase	Oxidoreductase in glycolysis and gluconeogenesis	AGGTCGGTGTGAACGGATTTG TGTAGACCATGTAGTTGAGGTCA	123	92.1
HPRT	Hypoxanthine guanine phosphoribosyl transferase	Purine metabolism	AAGCTTGCTGGTGAAAAGGA TTGCGCTCATCTTAGGCTTT	186	98.8
18S	18S ribosomal RNA	Ribosome RNA	TTGACTCAACACGGGAAACC AGACAAATCGCTCCACCAAC	121	102.5
RPLP0	Ribosomal protein, large, P0	Ribosomal proteins	AGATTCGGGATATGCTGTTGGC TCGGGTCCTAGACCAGTGTTC	109	92.4
B2M	Beta-2 microglobulin	Component of MHC class I	TTCTGGTGCTTGTCTCACTGA CAGTATGTTCGGCTTCCCATTC	104	97.7
TBP	TATA box-binding protein	Transcription factor	AGAACAATCCAGACTAGCAGCA GGGAACTTCACATCACAGCTC	120	95.4
PPIA	Peptidylprolyl isomerase A	Chaperone	GAGCTGTTTGCAGACAAAGTTC CCCTGGCACATGAATCCTGG	125	90.8
UBC	Ubiquitin C	Protein degradation	AGCCCAGTGTTACCACCAAGAAGG TCACACCCAAGAACAAGCACAAGGA	101	95.2
YWHAZ	Tyrosine 3-monooxygenase/tryptophan 5-monooxygenase activation protein, zeta polypeptide	Protein kinase C signaling pathway	GAAAAGTTCTTGATCCCCAATGC TGTGACTGGTCCACAATTCCTT	134	97.3

### Evaluation of Candidate Reference Genes

Reference gene expression variability was evaluated by a combined analysis of Delta-Ct method, Normfinder, geNorm, and BestKeeper. The Ct (cycle threshold) is defined as the number of cycles required for the fluorescent signal to cross the threshold (i.e., exceeds background level). Ct levels are inversely proportional to the amount of target nucleic acid in the sample (i.e., the lower the Ct level the greater the amount of target nucleic acid in the sample). Ct is specific to the expression of one gene whereas Delta Ct shows the difference of expression between two genes. This Delta-Ct method generated “pair of genes” comparisons between each candidate reference genes and the other candidate reference genes within each sample and calculated the average standard deviation (SD) against the other candidate reference genes (20). The NormFinder algorithm directly and robustly estimates candidate normalization gene expression stability and ranks reference genes depending on the variation within the intra- and the inter-group. As Andersen mentioned in his report, the NormFinder procedure focuses on differences between sample subgroups, and the result is less affected by the correlated expression of the reference genes (18). In 2002, Vandesompele et al. have developed the software GeNorm that evaluates the most stable pair of reference genes by the M value, which is calculated from the arithmetic mean of pair-wise variations of each gene, a low M value represented stable gene expression (11). BestKeeper calculates the expression variability of reference genes based on the SD, and the coefficient of variance (CV) takes into account Ct values of candidate reference genes instead of relative quantities (19). The genes with the lowest SD and CV were treated as the most stable reference genes, and reference genes with SD > 1 were excluded (19). Following these four analyses, each candidate reference gene obtained a specific ranking value. A consensual analysis was finally performed by the calculation of the geometric mean of the four ranking values for each gene leading to a consensus variability score for each reference gene.

### Statistical Analysis

All statistical analyses were conducted by SPSS 21.0. The Kolmogorov–Smirnov test was used to evaluate whether the data is normally distributed. We used the unpaired *t*-test for the normally distributed data and the Mann–Whitney *U*-test for the non-normally distributed data to calculate the difference between the NFD group and the HFD group, where *P* < 0.05 was considered statistically significant.

## Results

### Changes in Body Weight During the Development of Obesity

**Figure 1:**
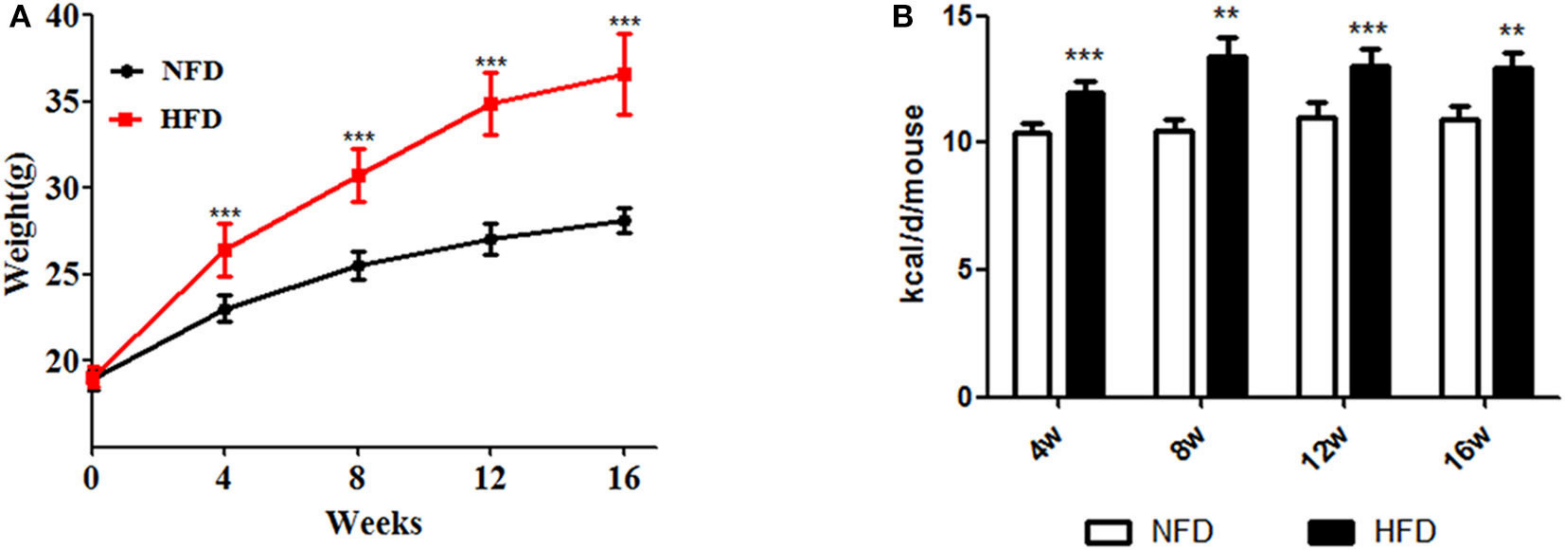
Changes in body weight and daily caloric intake during the development of obesity. Three- to four-week-old C57BL/6J male mice were fed a high-fat diet (HFD) for 4–16 weeks, with a normal-fat diet (NFD) as a control. **(A)** Body weight; **(B)** caloric intake. Data are shown as the means ± SD. **Compared to the NFD group, *P* < 0.01; ***compared to the NFD group, *P* < 0.005.

As shown in Figure 1, mouse body weight was significantly increased in the HFD group with more calories to intake, compared to the NFD group after feeding for 4, 8, 12, or 16 weeks (*P* < 0.05).

### Stability Analysis of Candidate Reference Genes

**Figure 2:**
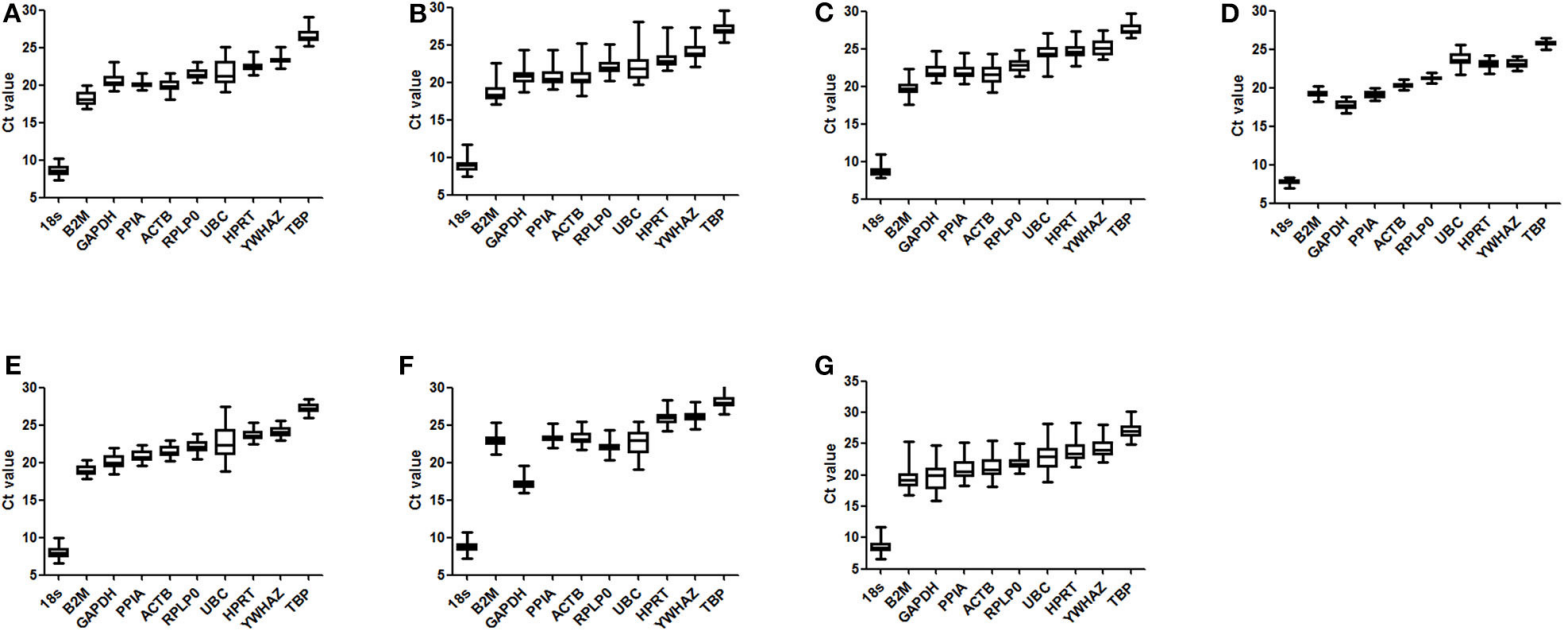
Distribution of Ct values for reference genes. Three- to four-week-old C57BL/6J male mice were fed a high-fat diet (HFD), with a normal-fat diet (NFD) as a control. At 4, 8, 12, and 16 weeks after feeding, mice were sacrificed respectively, and organs and tissues were dissected. The mRNA expression of reference genes was examined by RT-qPCR and their Ct values were analyzed by averaging the data in both groups of mice from the four time points. The boxes indicate the 25th and 75th percentiles, and the line across the box is the media, and whiskers correspond to the minimum and maximum values. **(A)** epididymal fat; **(B)** perirenal fat; **(C)** inguinal subcutaneous fat; **(D)** subscapular brown adipose tissue; **(E)** liver; **(F)** femoral muscle; **(G)** average for all types of tissues.

Figure 2 shows the profile and distribution of Ct values for the 10 candidate reference genes in different tissues. For each reference gene, similar expressional profiles were shown across samples in all types of tissues. However, a wide difference in expression levels was found among the 10 references, with the Ct values ranging from 8.47 ± 0.80 to 27.05 ± 1.02. The highest abundance gene was 18S RNA, which was significantly different from the others, whose abundance was in an increasing trend with B2M > GAPDH > PPIA > ACTB > RPLP0 > UBC > HPRT > YWHAZ > TBP. Meanwhile, some genes had a wide range in expression, e.g., B2M, GAPDH, ACTB, and UBC, indicating a higher variability, whereas others were in a narrow range, e.g., PPIA, RPLP0, and TBP, indicating more stably expressed.

To determine the ranking of the reference genes in tissues at different time points, the 10 candidate genes were analyzed by the geNorm and NormFinder algorithms, the comparative Delta-Ct method, and the BestKeeper software tool and further were calculated to identify stably expressed genes between the NFD group and the HFD group.

In the epididymal fat, as shown in Table 2, the identified set of four reference genes included PPIA, YWHAZ, RPLP0, and 18S after 4 weeks' feeding intervention. The optimal set of reference genes after 8 weeks' feeding appeared to be RPLP0, HPRT, B2M, and PPIA. The genes RPLP0, PPIA, B2M, and YWHAZ were represented a good choice as reference genes after 12 weeks' feeding. After 16 weeks' feeding, the best four reference genes were RPLP0, PPIA, HPRT, and YWHAZ. Furthermore, to assess the overall stability of each gene during the development of obesity, the data at the four time points were included in the analysis, and the result showed that PPIA, RPLP0, and YWHAZ were more stable in expression. In the perirenal fat, the calculation of the geometric mean from the NormFinder, GeNorm, BestKeeper, and Delta-Ct indentified RPLP0, PPIA, YWHAZ, ACTB, 18S, B2M, and TBP as more stably expressed at 4, 8, 12, or 16 weeks. Still, PPIA, RPLP0, and YWHAZ were shown as more stably expressed genes if all four time points were included for analysis (Table 3). In the inguinal fat, PPIA, TBP, YWHAZ, HPRT, RPLP0, and B2M were identified as more stably expressed at 4, 8, 12, or 16 weeks, and the expression of PPIA TBP and RPLP0 was indicated more stable with data from all four points analyzed (Table 4). In brown adipose tissue, PPIA, TBP, RPLP0, YWHAZ, and HRPT were more stably expressed at 4, 8, 12, or 16 weeks, and similar to the inguinal fat, RPLP0, TBP, and PPIA represented the best set of reference genes with all four time points considered (Table 5).

**Table 2:** Analysis of reference gene expression variability in the epididymal fat during the development of obesity.

	**NormFinder**		**GeNorm**		**BestKeeper**		**Delta-Ct**		**Consensus**	
	**Genes**	**Stability value**	**Genes**	**Stability value**	**Genes**	**SD**	**Genes**	**SD**	**Genes**	**Geometric mean of ranking values**
4 w NFD/HFD (*n* = 8/8)	RPLP0	0.135	PPIA	0.244	PPIA	0.260	YWHAZ	0.429	**PPIA**	1.57
	PPIA	0.139	YWHAZ	0.244	18S	0.286	RPLP0	0.430	**YWHAZ**	2.34
	ACTB	0.154	18S	0.259	YWHAZ	0.305	PPIA	0.430	**RPLP0**	2.66
	B2M	0.168	ACTB	0.298	ACTB	0.321	ACTB	0.467	**18S**	3.66
	YWHAZ	0.183	RPLP0	0.311	RPLP0	0.366	18S	0.509	ACTB	3.72
	18S	0.233	B2M	0.333	B2M	0.419	B2M	0.514	B2M	5.42
	HPRT	0.308	TBP	0.381	TBP	0.466	GAPDH	0.562	TBP	7.48
	TBP	0.372	GAPDH	0.409	GAPDH	0.500	TBP	0.566	GAPDH	7.97
	GAPDH	0.438	HPRT	0.477	HPRT	0.530	HPRT	0.667	HPRT	8.45
	UBC	0.452	UBC	0.575	UBC	0.767	UBC	0.882	UBC	10.00
8 w NFD/HFD (*n* = 8/8)	B2M	0.074	RPLP0	0.172	YWHAZ	0.163	RPLP0	0.309	**RPLP0**	2.00
	RPLP0	0.090	HPRT	0.172	18S	0.219	HPRT	0.321	**HPRT**	2.99
	PPIA	0.140	B2M	0.180	TBP	0.221	PPIA	0.335	**B2M**	3.03
	HPRT	0.149	TBP	0.202	PPIA	0.228	B2M	0.344	**PPIA**	3.83
	TBP	0.199	ACTB	0.223	HPRT	0.230	ACTB	0.345	YWHAZ	4.14
	YWHAZ	0.245	PPIA	0.236	ACTB	0.263	TBP	0.380	TBP	4.36
	ACTB	0.247	YWHAZ	0.247	B2M	0.265	YWHAZ	0.401	ACTB	5.69
	GAPDH	0.274	18S	0.275	RPLP0	0.287	GAPDH	0.421	18S	6.00
	18S	0.420	GAPDH	0.305	GAPDH	0.381	18S	0.495	GAPDH	8.49
	UBC	0.942	UBC	0.472	UBC	1.030	UBC	0.985	UBC	10.00
12 w NFD/HFD (*n* = 8/8)	PPIA	0.065	RPLP0	0.168	B2M	0.176	PPIA	0.313	**RPLP0**	1.68
	RPLP0	0.083	B2M	0.168	RPLP0	0.212	RPLP0	0.321	**PPIA**	1.86
	B2M	0.095	PPIA	0.198	18S	0.213	B2M	0.326	**B2M**	2.06
	YWHAZ	0.155	YWHAZ	0.220	PPIA	0.275	YWHAZ	0.379	**YWHAZ**	4.43
	HPRT	0.188	HPRT	0.250	GAPDH	0.281	GAPDH	0.382	18S	5.42
	18S	0.208	18S	0.272	YWHAZ	0.306	ACTB	0.385	HPRT	5.92
	GAPDH	0.267	GAPDH	0.297	HPRT	0.330	HPRT	0.405	GAPDH	5.92
	ACTB	0.282	TBP	0.313	ACTB	0.343	18S	0.416	ACTB	7.67
	TBP	0.321	ACTB	0.328	TBP	0.358	TBP	0.451	TBP	8.74
	UBC	0.376	UBC	0.445	UBC	0.636	UBC	0.788	UBC	10.00
16 w NFD/HFD (*n* = 8/8)	RPLP0	0.083	PPIA	0.152	TBP	0.146	PPIA	0.323	**RPLP0**	2.00
	HPRT	0.098	HPRT	0.152	RPLP0	0.230	RPLP0	0.351	**PPIA**	2.11
	B2M	0.112	B2M	0.194	GAPDH	0.253	YWHAZ	0.356	**HPRT**	3.36
	YWHAZ	0.125	RPLP0	0.232	PPIA	0.282	HPRT	0.364	**YWHAZ**	4.16
	PPIA	0.133	YWHAZ	0.244	YWHAZ	0.287	B2M	0.365	B2M	4.21
	18S	0.141	TBP	0.274	18S	0.298	GAPDH	0.402	TBP	4.28
	TBP	0.221	GAPDH	0.291	B2M	0.350	18S	0.407	GAPDH	5.63
	GAPDH	0.285	18S	0.303	HPRT	0.365	TBP	0.408	18S	6.70
	UBC	0.330	ACTB	0.342	ACTB	0.567	ACTB	0.491	ACTB	9.24
	ACTB	0.384	UBC	0.439	UBC	0.662	UBC	0.817	UBC	9.74
All NFD/HFD (*n* = 32/32)	PPIA	0.041	B2M	0.286	PPIA	0.293	RPLP0	0.488	**PPIA**	2.14
	RPLP0	0.047	ACTB	0.286	YWHAZ	0.324	YWHAZ	0.505	**RPLP0**	2.38
	YWHAZ	0.093	18S	0.343	HPRT	0.408	PPIA	0.542	**YWHAZ**	2.91
	B2M	0.111	RPLP0	0.358	RPLP0	0.527	ACTB	0.563	**B2M**	3.83
	HPRT	0.160	TBP	0.383	TBP	0.553	HPRT	0.568	HPRT	5.10
	TBP	0.213	YWHAZ	0.407	18S	0.561	B2M	0.598	ACTB	5.63
	18S	0.220	PPIA	0.421	ACTB	0.587	GAPDH	0.599	18S	5.80
	ACTB	0.231	GAPDH	0.439	GAPDH	0.619	TBP	0.611	TBP	5.89
	GAPDH	0.291	HPRT	0.465	B2M	0.621	18S	0.623	GAPDH	7.97
	UBC	0.372	UBC	0.742	UBC	1.366	UBC	1.508	UBC	10.00

*NFD, normal-fat diet; HFD, high-fat diet; All, all four points (4 w, 8 w, 12 w, 16 w); SD, standard deviation. Gene names indicated in bold are the most appropriate selection of 4 reference genes for all comparisons*.

**Table 3:** Analysis of reference gene expression variability in the perirenal fat during the development of obesity.

	**NormFinder**		**GeNorm**		**BestKeeper**		**Delta-Ct**		**Consensus**	
	**Genes**	**Stability value**	**Genes**	**Stability value**	**Genes**	**SD**	**Genes**	**SD**	**Genes**	**Geometric mean of ranking values**
4 w NFD/HFD (*n* = 8/8)	PPIA	0.054	TBP	0.179	18S	0.544	RPLP0	0.344	**RPLP0**	2.55
	RPLP0	0.110	YWHAZ	0.179	ACTB	0.577	PPIA	0.350	**PPIA**	2.83
	YWHAZ	0.119	RPLP0	0.200	B2M	0.589	ACTB	0.378	**YWHAZ**	3.13
	B2M	0.149	PPIA	0.224	YWHAZ	0.601	YWHAZ	0.388	**ACTB**	3.50
	ACTB	0.197	ACTB	0.255	TBP	0.632	TBP	0.431	TBP	3.50
	TBP	0.210	HPRT	0.282	HPRT	0.670	HPRT	0.434	18S	4.76
	HPRT	0.225	B2M	0.305	RPLP0	0.673	B2M	0.479	B2M	4.92
	18S	0.266	18S	0.323	PPIA	0.683	18S	0.508	HPRT	6.24
	GAPDH	0.415	GAPDH	0.397	GAPDH	0.986	GAPDH	0.617	GAPDH	9.00
	UBC	0.446	UBC	0.527	UBC	1.234	UBC	0.936	UBC	10.00
8 w NFD/HFD (*n* = 8/8)	RPLP0	0.095	RPLP0	0.242	18S	0.481	PPIA	0.404	**RPLP0**	2.00
	PPIA	0.134	PPIA	0.242	YWHAZ	0.537	RPLP0	0.416	**PPIA**	2.30
	YWHAZ	0.140	YWHAZ	0.289	ACTB	0.565	ACTB	0.470	**YWHAZ**	2.91
	B2M	0.221	18S	0.297	HPRT	0.574	YWHAZ	0.474	**18S**	3.31
	18S	0.251	HPRT	0.332	B2M	0.591	HPRT	0.486	ACTB	4.56
	TBP	0.306	ACTB	0.347	TBP	0.604	18S	0.491	HPRT	5.14
	HPRT	0.319	B2M	0.374	PPIA	0.608	TBP	0.526	B2M	5.79
	ACTB	0.397	TBP	0.397	RPLP0	0.634	B2M	0.545	TBP	6.70
	UBC	0.424	GAPDH	0.483	GAPDH	0.751	GAPDH	0.717	GAPDH	9.24
	GAPDH	0.642	UBC	0.582	UBC	1.053	UBC	0.871	UBC	9.74
12 w NFD/HFD (*n* = 8/8)	RPLP0	0.073	PPIA	0.102	TBP	0.401	PPIA	0.332	**PPIA**	1.97
	B2M	0.082	B2M	0.102	18S	0.402	RPLP0	0.388	**RPLP0**	2.06
	PPIA	0.104	RPLP0	0.158	RPLP0	0.483	B2M	0.402	**B2M**	2.63
	YWHAZ	0.215	HPRT	0.235	B2M	0.483	YWHAZ	0.470	**TBP**	3.94
	18S	0.217	YWHAZ	0.278	PPIA	0.484	TBP	0.506	YWHAZ	4.68
	TBP	0.294	ACTB	0.318	YWHAZ	0.533	HPRT	0.506	18S	4.86
	HPRT	0.344	18S	0.357	GAPDH	0.533	ACTB	0.515	HPRT	6.05
	UBC	0.454	TBP	0.386	HPRT	0.593	18S	0.540	ACTB	7.61
	ACTB	0.475	GAPDH	0.462	UBC	0.697	GAPDH	0.608	GAPDH	8.68
	GAPDH	0.600	UBC	0.550	ACTB	0.757	UBC	0.780	UBC	9.49
16 w NFD/HFD (*n* = 8/8)	PPIA	0.076	PPIA	0.196	TBP	0.661	PPIA	0.477	**PPIA**	1.57
	B2M	0.106	YWHAZ	0.196	18S	0.807	RPLP0	0.515	**RPLP0**	3.08
	RPLP0	0.170	RPLP0	0.239	GAPDH	0.887	YWHAZ	0.526	**YWHAZ**	3.13
	YWHAZ	0.228	B2M	0.301	YWHAZ	0.931	HPRT	0.546	**B2M**	4.09
	HPRT	0.252	HPRT	0.333	RPLP0	0.985	B2M	0.584	TBP	4.28
	18S	0.531	TBP	0.411	PPIA	0.988	GAPDH	0.690	18S	5.09
	GAPDH	0.572	18S	0.454	B2M	1.030	TBP	0.749	HPRT	5.32
	TBP	0.595	GAPDH	0.487	HPRT	1.134	18S	0.767	GAPDH	5.63
	ACTB	0.597	ACTB	0.561	ACTB	1.464	ACTB	0.787	ACTB	9.00
	UBC	1.033	UBC	0.762	UBC	1.822	UBC	1.347	UBC	10.00
All NFD/HFD (*n* = 32/32)	RPLP0	0.075	PPIA	0.294	TBP	0.637	RPLP0	0.503	**RPLP0**	1.86
	PPIA	0.077	YWHAZ	0.294	18S	0.688	PPIA	0.505	**PPIA**	2.21
	YWHAZ	0.096	HPRT	0.344	RPLP0	0.728	HPRT	0.544	**YWHAZ**	3.13
	B2M	0.102	RPLP0	0.389	YWHAZ	0.749	YWHAZ	0.561	**TBP**	3.98
	18S	0.122	B2M	0.419	B2M	0.771	B2M	0.606	HPRT	4.41
	HPRT	0.251	TBP	0.449	PPIA	0.799	TBP	0.635	18S	4.70
	TBP	0.329	18S	0.465	HPRT	0.806	18S	0.663	B2M	4.73
	ACTB	0.406	ACTB	0.513	GAPDH	0.815	ACTB	0.681	ACTB	8.24
	UBC	0.421	GAPDH	0.559	ACTB	0.868	GAPDH	0.694	GAPDH	8.97
	GAPDH	0.534	UBC	0.714	UBC	1.368	UBC	1.162	UBC	9.74

*NFD, normal-fat diet; HFD, high-fat diet; All, all four points (4 w, 8 w, 12 w, 16 w); SD, standard deviation. Gene names indicated in bold are the most appropriate selection of 4 reference genes for all comparisons*.

**Table 4:** Analysis of reference gene expression variability in the subcutaneous inguinal fat during the development of obesity.

	**NormFinder**		**GeNorm**		**BestKeeper**		**Delta-Ct**		**Consensus**	
	**Genes**	**Stability value**	**Genes**	**Stability value**	**Genes**	**SD**	**Genes**	**SD**	**Genes**	**Geometric mean of ranking values**
4 w NFD/HFD (*n* = 8/8)	TBP	0.066	PPIA	0.175	18S	0.413	YWHAZ	0.389	**PPIA**	2.55
	PPIA	0.123	YWHAZ	0.175	UBC	0.659	HPRT	0.396	**TBP**	2.78
	RPLP0	0.130	RPLP0	0.185	TBP	0.724	PPIA	0.399	**YWHAZ**	3.08
	HPRT	0.167	TBP	0.204	HPRT	0.737	RPLP0	0.401	**HPRT**	3.56
	YWHAZ	0.206	HPRT	0.237	B2M	0.786	TBP	0.404	RPLP0	4.12
	ACTB	0.260	B2M	0.260	ACTB	0.797	B2M	0.501	18S	5.62
	B2M	0.292	ACTB	0.295	PPIA	0.822	ACTB	0.519	B2M	5.96
	UBC	0.496	GAPDH	0.368	RPLP0	0.853	GAPDH	0.680	UBC	6.00
	GAPDH	0.615	UBC	0.462	YWHAZ	0.857	UBC	0.692	ACTB	6.48
	18S	0.777	18S	0.558	GAPDH	1.086	18S	0.941	GAPDH	8.71
8 w NFD/HFD (*n* = 8/8)	PPIA	0.107	RPLP0	0.159	18S	0.212	PPIA	0.322	**PPIA**	1.68
	TBP	0.123	YWHAZ	0.159	PPIA	0.363	YWHAZ	0.409	**RPLP0**	3.31
	HPRT	0.198	TBP	0.249	B2M	0.385	HPRT	0.424	**TBP**	3.31
	YWHAZ	0.214	PPIA	0.303	TBP	0.388	RPLP0	0.426	**YWHAZ**	3.46
	RPLP0	0.221	HPRT	0.332	HPRT	0.456	TBP	0.433	HPRT	3.87
	UBC	0.264	B2M	0.352	RPLP0	0.514	B2M	0.477	18S	4.60
	B2M	0.271	18S	0.384	GAPDH	0.523	UBC	0.517	B2M	5.24
	18S	0.306	UBC	0.415	UBC	0.523	18S	0.538	UBC	7.20
	ACTB	0.428	ACTB	0.456	YWHAZ	0.547	ACTB	0.557	GAPDH	9.15
	GAPDH	0.467	GAPDH	0.510	ACTB	0.735	GAPDH	0.608	ACTB	9.24
12 w NFD/HFD (*n* = 8/8)	HRRT	0.120	RPLP0	0.433	GAPDH	0.772	HRRT	0.629	**HRRT**	1.86
	PPIA	0.239	HPRT	0.433	B2M	0.776	PPIA	0.645	**PPIA**	2.45
	B2M	0.289	PPIA	0.492	PPIA	0.805	RPLP0	0.666	**RPLP0**	3.03
	RPLP0	0.331	B2M	0.516	YWHAZ	0.857	ACTB	0.719	**B2M**	3.46
	YWHAZ	0.354	ACTB	0.558	TBP	0.866	YWHAZ	0.726	YWHAZ	5.14
	18S	0.386	18S	0.584	HRRT	0.944	B2M	0.743	GAPDH	5.20
	ACTB	0.448	YWHAZ	0.603	RPLP0	0.976	TBP	0.805	ACTB	5.79
	TBP	0.505	TBP	0.629	ACTB	1.053	18S	0.807	TBP	6.88
	GAPDH	0.754	GAPDH	0.670	18S	1.054	GAPDH	0.879	18S	7.14
	UBC	1.015	UBC	0.869	UBC	2.011	UBC	1.478	UBC	10.00
16 w NFD/HFD (*n* = 8/8)	PPIA	0.099	B2M	0.174	18S	0.326	PPIA	0.403	**PPIA**	2.11
	RPLP0	0.195	HPRT	0.174	GAPDH	0.502	YWHAZ	0.467	**RPLP0**	3.08
	YWHAZ	0.205	YWHAZ	0.255	RPLP0	0.631	RPLP0	0.470	**YWHAZ**	3.22
	TBP	0.228	PPIA	0.296	TBP	0.720	HPRT	0.472	**HPRT**	4.23
	HPRT	0.283	RPLP0	0.331	PPIA	0.759	TBP	0.488	B2M	4.24
	B2M	0.363	TBP	0.344	YWHAZ	0.829	B2M	0.538	TBP	4.68
	UBC	0.412	ACTB	0.401	UBC	0.963	ACTB	0.598	18S	5.33
	ACTB	0.541	GAPDH	0.475	HPRT	0.967	GAPDH	0.621	GAPDH	5.83
	GAPDH	0.552	18S	0.533	B2M	1.008	18S	0.768	ACTB	7.91
	18S	0.572	UBC	0.588	ACTB	1.180	UBC	0.776	UBC	8.37
All NFD/HFD (*n* = 32/32)	PPIA	0.085	PPIA	0.358	TBP	0.710	PPIA	0.563	**PPIA**	1.19
	B2M	0.112	TBP	0.358	PPIA	0.730	RPLP0	0.651	**TBP**	2.45
	RPLP0	0.135	RPLP0	0.408	18S	0.730	TBP	0.681	**RPLP0**	2.91
	HPRT	0.144	HPRT	0.485	RPLP0	0.798	HPRT	0.682	**B2M**	4.16
	YWHAZ	0.176	B2M	0.530	B2M	0.801	ACTB	0.708	HPRT	4.60
	TBP	0.193	ACTB	0.567	GAPDH	0.833	B2M	0.739	18S	6.59
	18S	0.263	YWHAZ	0.619	HPRT	0.860	YWHAZ	0.802	YWHAZ	6.65
	ACTB	0.368	GAPDH	0.680	YWHAZ	0.901	GAPDH	0.843	ACTB	6.82
	UBC	0.471	18S	0.762	ACTB	0.980	UBC	1.015	GAPDH	7.87
	GAPDH	0.575	UBC	0.832	UBC	1.068	18S	1.072	UBC	9.49

*NFD, normal-fat diet; HFD, high-fat diet; All, all four points (4 w, 8 w, 12 w, 16 w); SD, standard deviation. Gene names indicated in bold are the most appropriate selection of 4 reference genes for all comparisons*.

**Table 5:** Analysis of reference gene expression variability in the brown adipose tissue during the development of obesity.

	**NormFinder**		**GeNorm**		**BestKeeper**		**Delta-Ct**		**Consensus**	
	**Genes**	**Stability value**	**Genes**	**Stability value**	**Genes**	**SD**	**Genes**	**SD**	**Genes**	**Geometric mean of ranking values**
4 w NFD/HFD (*n* = 8/8)	PPIA	0.056	PPIA	0.136	PPIA	0.120	PPIA	0.266	**PPIA**	1.00
	RPLP0	0.095	TBP	0.136	TBP	0.139	TBP	0.288	**TBP**	2.38
	YWHAZ	0.123	ACTB	0.165	RPLP0	0.149	YWHAZ	0.304	**RPLP0**	3.13
	TBP	0.124	RPLP0	0.180	ACTB	0.152	RPLP0	0.323	**YWHAZ**	4.24
	ACTB	0.194	18S	0.201	18S	0.165	ACTB	0.323	ACTB	4.36
	18S	0.195	YWHAZ	0.219	YWHAZ	0.237	18S	0.336	18S	5.23
	UBC	0.210	UBC	0.262	UBC	0.283	UBC	0.388	UBC	7.00
	HPRT	0.221	HPRT	0.296	B2M	0.319	HPRT	0.426	HPRT	8.24
	B2M	0.222	B2M	0.326	HPRT	0.337	B2M	0.440	B2M	8.74
	GAPDH	0.362	GAPDH	0.366	GAPDH	0.426	GAPDH	0.527	GAPDH	10.00
8 w NFD/HFD (*n* = 8/8)	PPIA	0.073	PPIA	0.187	PPIA	0.152	YWHAZ	0.295	**PPIA**	1.19
	YWHAZ	0.082	TBP	0.187	RPLP0	0.181	PPIA	0.311	**YWHAZ**	2.38
	RPLP0	0.092	RPLP0	0.189	HPRT	0.181	HPRT	0.321	**RPLP0**	2.91
	HPRT	0.139	YWHAZ	0.216	YWHAZ	0.184	RPLP0	0.335	**HPRT**	3.66
	TBP	0.145	HPRT	0.239	18S	0.206	TBP	0.363	TBP	4.33
	B2M	0.189	GAPDH	0.262	B2M	0.207	GAPDH	0.408	B2M	6.70
	18S	0.211	ACTB	0.288	TBP	0.207	B2M	0.408	GAPDH	7.14
	GAPDH	0.249	B2M	0.305	ACTB	0.211	ACTB	0.426	18S	7.30
	ACTB	0.267	18S	0.332	GAPDH	0.294	18S	0.466	ACTB	7.97
	UBC	0.332	UBC	0.419	UBC	0.492	UBC	0.714	UBC	10.00
12 w NFD/HFD (*n* = 8/8)	RPLP0	0.079	RPLP0	0.209	PPIA	0.119	YWHAZ	0.281	**RPLP0**	1.41
	YWHAZ	0.109	TBP	0.209	RPLP0	0.138	RPLP0	0.284	**PPIA**	2.45
	PPIA	0.119	18S	0.218	HPRT	0.150	PPIA	0.296	**YWHAZ**	2.63
	HPRT	0.127	PPIA	0.236	YWHAZ	0.165	HPRT	0.297	**HPRT**	3.94
	18S	0.153	HPRT	0.252	18S	0.180	TBP	0.323	TBP	4.36
	TBP	0.189	YWHAZ	0.262	TBP	0.185	18S	0.340	18S	4.61
	B2M	0.198	ACTB	0.275	ACTB	0.198	B2M	0.352	B2M	7.48
	ACTB	0.280	B2M	0.287	B2M	0.229	ACTB	0.367	ACTB	7.48
	UBC	0.308	UBC	0.324	GAPDH	0.361	UBC	0.427	UBC	9.24
	GAPDH	0.362	GAPDH	0.354	UBC	0.377	GAPDH	0.473	GAPDH	9.74
16 w NFD/HFD (*n* = 8/8)	RPLP0	0.036	RPLP0	0.151	RPLP0	0.090	RPLP0	0.314	**RPLP0**	1.00
	YWHAZ	0.106	HPRT	0.151	YWHAZ	0.117	YWHAZ	0.317	**YWHAZ**	2.00
	HPRT	0.111	HPRT	0.176	HPRT	0.120	HPRT	0.320	**HPRT**	3.00
	PPIA	0.113	PPIA	0.187	PPIA	0.138	PPIA	0.347	**PPIA**	4.00
	18S	0.130	TBP	0.227	TBP	0.188	TBP	0.398	TBP	5.44
	B2M	0.200	18S	0.261	18S	0.200	18S	0.413	18S	5.73
	TBP	0.227	ACTB	0.297	B2M	0.250	ACTB	0.462	B2M	7.20
	UBC	0.291	B2M	0.323	ACTB	0.296	B2M	0.468	ACTB	7.71
	ACTB	0.322	GAPDH	0.367	GAPDH	0.360	GAPDH	0.547	GAPDH	9.24
	GAPDH	0.408	UBC	0.430	UBC	0.465	UBC	0.669	UBC	9.46
All NFD/HFD (*n* = 32/32)	RPLP0	0.062	RPLP0	0.236	RPLP0	0.152	RPLP0	0.427	**RPLP0**	1.00
	PPIA	0.080	TBP	0.236	18S	0.193	PPIA	0.442	**TBP**	3.22
	YWHAZ	0.107	ACTB	0.288	TBP	0.215	TBP	0.461	**PPIA**	3.60
	HPRT	0.146	18S	0.329	ACTB	0.243	YWHAZ	0.466	**18S**	4.23
	18S	0.149	B2M	0.370	B2M	0.324	HPRT	0.477	ACTB	5.05
	TBP	0.155	PPIA	0.407	GAPDH	0.403	ACTB	0.495	YWHAZ	5.09
	B2M	0.156	YWHAZ	0.423	PPIA	0.423	B2M	0.500	B2M	5.92
	UBC	0.253	HPRT	0.435	YWHAZ	0.423	18S	0.523	HPRT	6.16
	ACTB	0.259	GAPDH	0.467	HPRT	0.445	GAPDH	0.604	GAPDH	8.35
	GAPDH	0.347	UBC	0.525	UBC	0.627	UBC	0.732	UBC	9.46

*NFD, normal-fat diet; HFD, high-fat diet; All, all four points (4 w, 8 w, 12 w, 16 w); SD, standard deviation. Gene names indicated in bold are the most appropriate selection of 4 reference genes for all comparisons*.

In the liver, as shown in Table 6, the more stable reference genes were B2M, RPLP0, PPIA, and ACTB at 4 weeks, HRPT, PPIA, YWHAZ, and RPLP0 at 8 weeks, PPIA, RPLP0, HRPT, and TBP at 12 weeks, and YWHAZ, RPLP0, PPIA, and HRPT at 16 weeks. The expressions of HRPT, YWHAZ, and RPLP0 were identified to be more stable for all the four time points.

**Table 6:** Analysis of reference gene expression variability in the liver during the development of obesity.

	**NormFinder**		**GeNorm**		**BestKeeper**		**Delta-Ct**		**Consensus**	
	**Genes**	**Stability value**	**Genes**	**Stability value**	**Genes**	**SD**	**Genes**	**SD**	**Genes**	**Geometric mean of ranking values**
4 w NFD/HFD (*n* = 8/8)	B2M	0.047	PPIA	0.179	18S	0.255	RPLP0	0.299	**B2M**	2.00
	RPLP0	0.052	B2M	0.179	B2M	0.353	PPIA	0.328	**RPLP0**	2.21
	YWHAZ	0.059	RPLP0	0.183	TBP	0.377	ACTB	0.340	**PPIA**	2.66
	ACTB	0.075	YWHAZ	0.198	RPLP0	0.383	B2M	0.347	**ACTB**	4.36
	PPIA	0.079	ACTB	0.205	PPIA	0.453	YWHAZ	0.356	YWHAZ	4.68
	HPRT	0.107	TBP	0.223	ACTB	0.457	TBP	0.406	18S	5.20
	TBP	0.110	GAPDH	0.238	GAPDH	0.462	HPRT	0.417	TBP	5.24
	GAPDH	0.142	HPRT	0.259	YWHAZ	0.502	GAPDH	0.423	HPRT	7.42
	18S	0.234	18S	0.345	HPRT	0.529	18S	0.716	GAPDH	7.48
	UBC	0.334	UBC	0.470	UBC	1.061	UBC	0.874	UBC	10.00
8 w NFD/HFD (*n* = 8/8)	YWHAZ	0.047	PPIA	0.116	18S	0.372	HPRT	0.328	**HPRT**	2.21
	PPIA	0.052	HPRT	0.116	TBP	0.397	RPLP0	0.343	**PPIA**	2.55
	HPRT	0.066	YWHAZ	0.136	B2M	0.438	PPIA	0.351	**YWHAZ**	3.31
	RPLP0	0.079	RPLP0	0.168	HPRT	0.474	ACTB	0.365	**RPLP0**	3.72
	ACTB	0.165	GAPDH	0.192	ACTB	0.478	YWHAZ	0.371	ACTB	4.95
	GAPDH	0.179	ACTB	0.207	RPLP0	0.498	B2M	0.412	18S	5.20
	B2M	0.196	B2M	0.219	PPIA	0.510	GAPDH	0.429	B2M	5.45
	TBP	0.307	TBP	0.255	YWHAZ	0.541	TBP	0.505	TBP	5.66
	18S	0.553	18S	0.312	GAPDH	0.604	18S	0.650	GAPDH	6.59
	UBC	0.844	UBC	0.539	UBC	1.377	UBC	1.258	UBC	10.00
12 w NFD/HFD (*n* = 8/8)	PPIA	0.056	PPIA	0.140	18S	0.170	RPLP0	0.316	**PPIA**	1.86
	B2M	0.082	HPRT	0.140	YWHAZ	0.295	PPIA	0.321	**RPLP0**	3.08
	RPLP0	0.095	TBP	0.154	HPRT	0.308	HPRT	0.328	**HPRT**	3.22
	TBP	0.117	YWHAZ	0.169	TBP	0.323	TBP	0.328	**TBP**	3.72
	ACTB	0.137	B2M	0.180	RPLP0	0.360	B2M	0.332	YWHAZ	4.28
	HPRT	0.142	RPLP0	0.202	PPIA	0.395	YWHAZ	0.337	B2M	4.33
	YWHAZ	0.148	ACTB	0.227	B2M	0.396	ACTB	0.365	18S	5.20
	GAPDH	0.280	GAPDH	0.255	ACTB	0.426	GAPDH	0.429	ACTB	6.65
	18S	0.580	18S	0.328	GAPDH	0.561	18S	0.661	GAPDH	8.24
	UBC	0.637	UBC	0.439	UBC	0.939	UBC	0.796	UBC	10.00
16 w NFD/HFD (*n* = 8/8)	PPIA	0.064	RPLP0	0.045	HPRT	0.466	YWHAZ	0.278	**YWHAZ**	2.000
	YWHAZ	0.065	YWHAZ	0.045	TBP	0.471	RPLP0	0.283	**RPLP0**	2.340
	RPLP0	0.085	PPIA	0.097	B2M	0.473	PPIA	0.294	**PPIA**	2.913
	B2M	0.156	HPRT	0.146	YWHAZ	0.576	HPRT	0.330	**HPRT**	2.991
	HPRT	0.175	B2M	0.174	RPLP0	0.585	B2M	0.340	B2M	4.162
	TBP	0.239	TBP	0.191	18S	0.603	TBP	0.354	TBP	4.559
	ACTB	0.272	ACTB	0.214	ACTB	0.619	ACTB	0.374	ACTB	7.000
	18S	0.279	18S	0.232	PPIA	0.620	18S	0.391	18S	7.445
	GAPDH	0.318	GAPDH	0.276	GAPDH	0.677	GAPDH	0.455	GAPDH	9.000
	UBC	0.633	UBC	0.399	UBC	1.183	UBC	0.887	UBC	10.000
All NFD/HFD (*n* = 32/32)	RPLP0	0.032	YWHAZ	0.195	TBP	0.480	HPRT	0.430	**HPRT**	2.21
	PPIA	0.033	HPRT	0.195	HPRT	0.502	RPLP0	0.440	**YWHAZ**	2.59
	B2M	0.103	B2M	0.258	YWHAZ	0.514	YWHAZ	0.440	**RPLP0**	3.25
	ACTB	0.105	TBP	0.271	B2M	0.549	PPIA	0.475	**TBP**	3.74
	YWHAZ	0.109	PPIA	0.295	18S	0.589	ACTB	0.481	B2M	3.83
	HPRT	0.156	ACTB	0.311	PPIA	0.614	B2M	0.509	PPIA	3.94
	TBP	0.161	RPLP0	0.319	ACTB	0.657	TBP	0.538	ACTB	5.38
	GAPDH	0.240	GAPDH	0.333	RPLP0	0.663	GAPDH	0.561	18S	7.77
	18S	0.402	18S	0.393	GAPDH	0.723	18S	0.731	GAPDH	8.24
	UBC	0.543	UBC	0.667	UBC	1.849	UBC	1.463	UBC	10.00

*NFD, normal-fat diet; HFD, high-fat diet; All, all four points (4 w, 8 w, 12 w, 16 w); SD, standard deviation. Gene names indicated in bold are the most appropriate selection of 4 reference genes for all comparisons*.

In femoral muscle, as shown in Table 7, the more stable reference genes were RPLP0, HRPT, PPIA, and TBP at 4 weeks, HRPT, PPIA, YWHAZ, and GAPDH at 8 weeks, YWHAZ, TBP, PPIA, and HRPT at 12 weeks, and RPLP0, YWHAZ, B2M, and PPIA at 16 weeks. If all data from the four time points were analyzed, YWHAZ, RPLP0, and GAPDH were shown more stable in expression.

**Table 7:** Analysis of reference gene expression variability in the femoral muscle during the development of obesity.

	**NormFinder**		**GeNorm**		**BestKeeper**		**Delta-Ct**		**Consensus**	
	**Genes**	**Stability value**	**Genes**	**Stability value**	**Genes**	**SD**	**Genes**	**SD**	**Genes**	**Geometric mean of ranking values**
4 w NFD/HFD (*n* = 8/8)	PPIA	0.024	HPRT	0.196	RPLP0	0.253	RPLP0	0.328	**RPLP0**	2.11
	HPRT	0.035	TBP	0.196	PPIA	0.304	HPRT	0.374	**HPRT**	2.21
	TBP	0.045	YWHAZ	0.208	TBP	0.312	PPIA	0.379	**PPIA**	2.34
	YWHAZ	0.056	RPLP0	0.216	18S	0.321	TBP	0.385	**TBP**	2.91
	RPLP0	0.066	PPIA	0.238	GAPDH	0.333	YWHAZ	0.386	YWHAZ	4.53
	B2M	0.087	ACTB	0.262	HPRT	0.343	ACTB	0.422	ACTB	6.70
	ACTB	0.106	GAPDH	0.291	YWHAZ	0.344	B2M	0.432	18S	6.90
	18S	0.114	B2M	0.315	ACTB	0.367	GAPDH	0.483	GAPDH	7.09
	GAPDH	0.132	18S	0.334	B2M	0.379	18S	0.496	B2M	7.42
	UBC	0.406	UBC	0.504	UBC	1.056	UBC	1.082	UBC	10.00
8 w NFD/HFD (*n* = 8/8)	HPRT	0.127	HPRT	0.191	18S	0.499	PPIA	0.355	**HPRT**	2.43
	TBP	0.135	GAPDH	0.191	B2M	0.565	RPLP0	0.368	**PPIA**	3.22
	GAPDH	0.148	PPIA	0.230	YWHAZ	0.619	YWHAZ	0.394	**YWHAZ**	3.46
	YWHAZ	0.159	YWHAZ	0.260	RPLP0	0.633	B2M	0.396	**GAPDH**	3.98
	ACTB	0.166	TBP	0.280	HPRT	0.685	ACTB	0.405	RPLP0	4.28
	PPIA	0.168	RPLP0	0.295	PPIA	0.693	GAPDH	0.406	B2M	4.60
	RPLP0	0.213	B2M	0.308	GAPDH	0.715	HPRT	0.407	TBP	5.03
	B2M	0.334	ACTB	0.320	TBP	0.728	TBP	0.444	18S	5.20
	18S	0.464	18S	0.352	ACTB	0.824	18S	0.575	ACTB	6.51
	UBC	1.027	UBC	0.525	UBC	1.449	UBC	1.034	UBC	10.00
12 w NFD/HFD (*n* = 8/8)	TBP	0.085	YWHAZ	0.237	TBP	0.370	PPIA	0.332	**YWHAZ**	2.63
	HPRT	0.119	18S	0.237	YWHAZ	0.376	RPLP0	0.402	**TBP**	2.71
	PPIA	0.127	PPIA	0.255	HPRT	0.383	ACTB	0.406	**PPIA**	2.71
	B2M	0.134	GAPDH	0.268	B2M	0.385	YWHAZ	0.408	**HPRT**	4.41
	GAPDH	0.165	RPLP0	0.280	18S	0.404	B2M	0.415	18S	4.86
	YWHAZ	0.182	ACTB	0.295	PPIA	0.423	TBP	0.430	B2M	5.03
	18S	0.247	HPRT	0.320	ACTB	0.428	GAPDH	0.443	RPLP0	5.18
	ACTB	0.292	B2M	0.335	RPLP0	0.460	18S	0.450	ACTB	5.63
	RPLP0	0.333	TBP	0.386	GAPDH	0.472	HPRT	0.457	GAPDH	5.96
	UBC	0.875	UBC	0.608	UBC	1.037	UBC	1.183	UBC	10.00
16 w NFD/HFD (*n* = 8/8)	RPLP0	0.136	RPLP0	0.217	PPIA	0.273	RPLP0	0.339	**RPLP0**	1.68
	B2M	0.176	YWHAZ	0.217	TBP	0.515	YWHAZ	0.414	**YWHAZ**	2.63
	YWHAZ	0.179	B2M	0.255	18S	0.560	B2M	0.414	**B2M**	3.08
	GAPDH	0.188	ACTB	0.273	YWHAZ	0.592	GAPDH	0.450	**PPIA**	4.56
	ACTB	0.195	GAPDH	0.294	B2M	0.596	ACTB	0.469	GAPDH	4.68
	PPIA	0.269	18S	0.331	GAPDH	0.614	HPRT	0.513	18S	5.45
	18S	0.311	HPRT	0.366	UBC	0.626	18S	0.521	ACTB	5.62
	TBP	0.318	TBP	0.396	RPLP0	0.634	PPIA	0.538	TBP	5.83
	HPRT	0.349	PPIA	0.440	HPRT	0.654	TBP	0.543	HPRT	7.64
	UBC	0.499	UBC	0.540	ACTB	0.738	UBC	0.811	UBC	9.15
All NFD/HFD (*n* = 32/32)	GAPDH	0.062	HPRT	0.278	PPIA	0.431	RPLP0	0.403	**YWHAZ**	2.21
	YWHAZ	0.090	TBP	0.278	YWHAZ	0.512	YWHAZ	0.460	**RPLP0**	2.63
	TBP	0.108	YWHAZ	0.318	RPLP0	0.557	ACTB	0.462	**GAPDH**	3.64
	RPLP0	0.117	RPLP0	0.331	18S	0.559	B2M	0.490	**HPRT**	3.66
	HPRT	0.126	GAPDH	0.338	TBP	0.592	GAPDH	0.494	TBP	3.81
	PPIA	0.136	ACTB	0.359	HPRT	0.594	HPRT	0.499	PPIA	4.56
	B2M	0.144	B2M	0.387	GAPDH	0.615	TBP	0.502	ACTB	6.00
	ACTB	0.150	18S	0.410	B2M	0.630	PPIA	0.506	B2M	6.29
	18S	0.279	PPIA	0.433	ACTB	0.670	18S	0.593	18S	7.14
	UBC	0.419	UBC	0.609	UBC	1.289	UBC	1.101	UBC	10.00

*NFD, normal-fat diet; HFD, high-fat diet; All, all four points (4 w, 8 w, 12 w, 16 w); SD, standard deviation. Gene names indicated in bold are the most appropriate selection of 4 reference genes for all comparisons*.

As shown in Tables 2–7, UBC was the least stable gene in expression in all six types of tissues during the development of obesity, followed by GAPDH, ACTB, and 18S, which have been commonly used in the adipose tissue and hepatic tissue.

### Effect of High-Fat Diet on Reference Gene Expression

Furthermore, the candidate genes were examined by using the top two stable reference genes as internal standards, and similar results were shown that PPIA and RPLP0 in all four types of adipose tissue, HRPT, YWHAZ, and RPLP0 in the liver, and YWHAZ, RPLP0, and GAPDH in muscle were more stably expressed from 4 to 16 weeks. The more changeable expression was seen with UBC in all tissues, and ACTB and GAPDH in the four types of adipose tissue and the liver.

**Figure 3:**
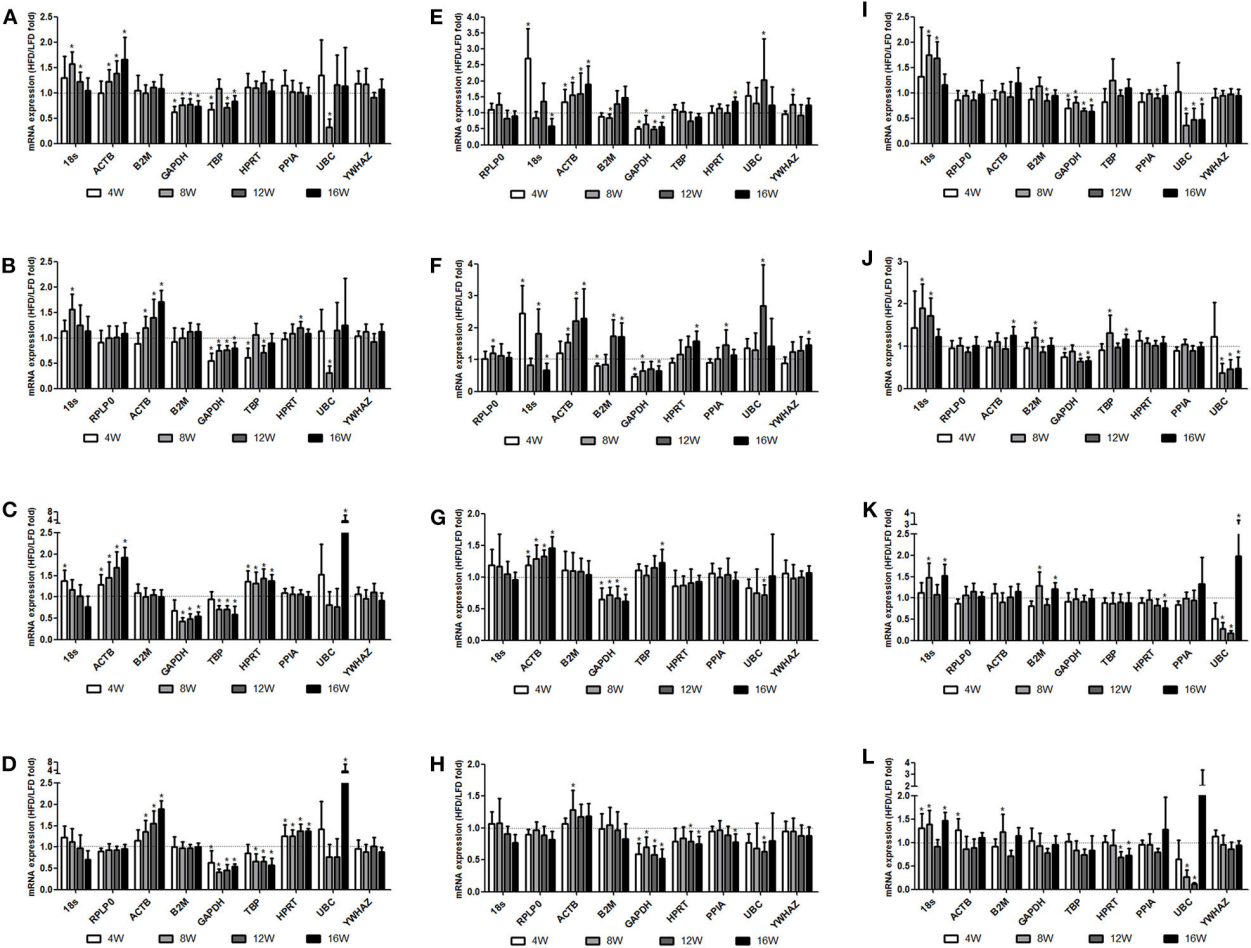
Changes in the mRNA expression of reference genes in tissues during the process of obesity. Three- to four-week-old C57BL/6J male mice were fed a high-fat diet (HFD), with a normal-fat diet (NFD) as a control. At 4, 8, 12, and 16 weeks after feeding, mice were sacrificed respectively, and organs and tissues were dissected. The mRNA expression of reference genes was examined by RT-qPCR. The relative expression of reference genes with the HFD feeding to the NFD feeding was determined with the top two candidate reference genes as the invariant internal control in each tissue, specifically normalized to RPLP0 **(A)** or PPIA **(B)** in the epididymal fat, RPLP0 **(C)** or PPIA **(D)** in the perirenal fat, PPIA **(E)** or TBP **(F)** in the subcutaneous inguinal fat, RPLP0 **(G)** or TBP **(H)** in the subscapular brown adipose tissue, HPRT **(I)** or YWHAZ **(J)** in the liver, and YWHAZ **(K)** or RPLP0 **(L)** in the femoral muscle. All data are presented as the means ± SD; *n* = 8 in either the HFD or the NFD group at each time points. *Significantly different from the NFD group (*p* < 0.05).

We found that HFD feeding increased the mRNA levels of ACTB in four types of adipose tissues at 4w, 8w, 12w, and 16w, and the differences became more significant with the development of obesity. The GAPDH mRNA levels were decreased in four types of adipose tissues and liver at 4w, 8w, 12w, and 16w after HFD feeding (Figure 3, Supplementary Tables 3–8). Significant changes were found in the TNFα (a positive control) expression with HFD feeding (Supplementary Figure 3).

## Discussion

In this study, a detailed analysis of candidate reference genes in the fat (epididymal, perirenal, subcutaneous inguinal, and brown adipose tissue), liver, and femoral muscle at different time points (4, 8, 12, and 16 weeks), demonstrated a set of more stable reference genes, which were more suitable for use in energy and fat metabolism associated tissues in the HFD induced-obese mice. Although the four methods, which were applied for analysis of gene stability, use different analytical approaches, the results were similar in all groups. The most stable reference genes were slightly different for a specific organ or tissue in a specific time point during the process of obesity pathogenesis. Further analysis with combined time points indicated that the genes PPIA, RPLP0, and YWHAZ were ranked top three among the 10 reference genes in the epididymal fat and the perirenal fat and that PPIA, TBP, and RPLP0 were ranked top three in the inguinal fat and brown adipose tissue. In the liver, the top three more stably expressed genes were HRPT, YWHAZ, and RPLP0, and in the femoral muscle, YWHAZ, RPLP0, and GAPDH were identified as the top three genes.

Reference genes have been demonstrated to be variable in obesity by several studies. Being consistent with our findings, RPLP0 has been validated as one of the top-ranking reference genes in human and rat adipose tissue (16, 23). TBP and ATPF1 should be used as reference genes in qPCR experiments on the adipose tissue with metabolic disease (7). In the DIO mouse model, the most stable candidates are 18S and PPIA in the epididymal fat and HPRT1 and PPIA in the heart, whereas they are 18S and GAPDH in the epididymal fat and RPI7 and GAPDH in the heart in wild-type and db/db mice (15). There have been quantities of studies on HFD-induced obesity, especially in those metabolism-related tissues, such as adipose tissues, liver, and muscle (24, 25). For example, Zhang et al. (23) found that four frequently used reference genes have different expression stabilities in three types of adipose tissue from the control and high-fat diet rats. Perez and his colleagues assessed the relative stability of the 10 candidate reference genes in perigonadal adipose tissue from chow and high-fat high-sucrose-fed C57BL/6 mice (15). Our results are consistent with a previous study by Zhang et al., who validated that RPLP0 was the best reference gene in three types of rat adipose tissue (23). In another study, Gabrilsson and colleagues evaluated reference genes in human adipose tissue. They found that of the frequently used reference genes, RPLP0 was highest ranked (16). In this study, different reference genes were validated for each time point in liver (B2M for 4 w, HPRT for 8 w, PPIA for 12 w, YWHAZ for 16 w) and femoral muscle (HPRT for 8 w, YWHAZ for 12 w, PPIA for 4w, and 16 w).

The genes ACTB, GAPDH, and 18S have been mostly employed as the sole reference genes for qPCR data normalization (26–31). However, our results showed that they were more unstable genes in the adipose and hepatic tissues. In consistent with our findings, it has been reported that ACTB is one of the most unstable genes in mouse models of obesity and diabetes and that the ACTB expression in the hypothalamus and intestine from an obese rat model is markedly altered with changes in energy status (32). Also, the expressions of B2M, GAPDH, and ACTB are varied with types of adipose tissue, metabolic status, and different experimental conditions in mice (7, 33). Regarding the expression of GAPDH and ACTB in human tissues, the controversy exists. Some studies reported that GAPDH and ACTB are reported to be among the most stably expressed in the human samples (12, 13). Mehta et al. validated ACTB as a stable reference gene most suitable for gene expression studies of human visceral adipose tissue (13), and GAPDH, together with CYCA and RPL27, has been identified as the most stable genes in human epicardial fat depots of lean, overweight, and obese subjects (17), whereas others demonstrated that SDHA and HSPCB are ranked as the most stable candidates in human in the subcutaneous fat (15), and GAPDH and ACTB showed a significant variation in human adipose (16) and are less appropriate reference genes in human omental and subcutaneous adipose tissue from obesity and type 2 diabetes patients, because of the variational expression (34). Thus, the stability of expression of reference genes differs between species and between healthy/disordered tissue within one specie.

It is noteworthy that reference proteins, also called housekeeping proteins, are key internal controls serving to normalize the western blot or immunoblot data. In studies on obesity and other diseases, GAPDH, β-actin, or β-tubulin has been used extensively as housekeeping protein in determination of target protein expression. However, it has been previously reported that common housekeeping proteins are not always reliable loading controls (35, 36). Although a direct correlation between the levels of mRNA and that of protein exists due to the expressed mRNA translated into protein (37), many studies have demonstrated discrepancies between mRNA and protein levels, indicating that mRNA levels are not sufficient to predict protein levels in many scenarios (38–40). Therefore, the results from reference genes cannot be extrapolated to reference proteins. With regard to the more stable reference genes screened in the current study, their translated proteins as reference controls have been investigated by few studies. Kim et al. reported that among the seven housekeeping proteins (HPRT1, PPIA, GYS1, TBP, YWHAZ, GAPDH, and ACTB) in the rat cerebrum, cerebellum, cardiac ventricle, and atrium, psoas major muscle, femoral muscle, liver, spleen, kidney, and aorta tissues, HPRT1, PPIA, YWHAZ, and GAPDH are more stably expressed across tissues (41). Nonetheless, whether the corresponding proteins translated from more stably expressed genes PPIA, RPLP0, and YWHAZ are appropriate for references in protein studies of obesity, needs to be clarified in the future.

In conclusion, although the most stable reference gene was different among specific organs/tissues with the development of obesity, PPIA, RPLP0, or YWHAZ should be used as reference gene in qPCR experiments on adipose, hepatic tissues, and muscles of mice in diet-induced obesity and associated metabolic complications.

## Data Availability Statement

The original contributions presented in the study are included in the article/Supplementary Material, further inquiries can be directed to the corresponding author.

## Ethics Statement

The animal study was reviewed and approved by Committee on the Ethics of Institute of Laboratory Animal Sciences, National Institute of Occupational Health and Poison Control of China.

## Author Contributions

XF participated in the study design, statistical analysis, and paper writing. HY carried out mouse feeding and the mRNA expression experiments. XL and QS participated in the mRNA expression experiments. LL, PL, RW, and TT participated in the statistical analysis. KQ conceived the study and participated in its design and coordination. The paper was written by XF and KQ. All authors reviewed and commented on the manuscript.

## Conflict of Interest

LL was employed by the company Zeesan Biotech. The remaining authors declare that the research was conducted in the absence of any commercial or financial relationships that could be construed as a potential conflict of interest.
