# Supplementary material for: High-Fat Diet Alters the Expression of Reference Genes in Male Mice
Source: Front Nutr. 2020 Nov 24;7:589771. doi: 10.3389/fnut.2020.589771 (PMC7732482; doi:10.3389/fnut.2020.589771)
Supplement: Supplementary file 1 [file Table_1.DOCX]

Supplementary Materials

## Supplementary Figures

**
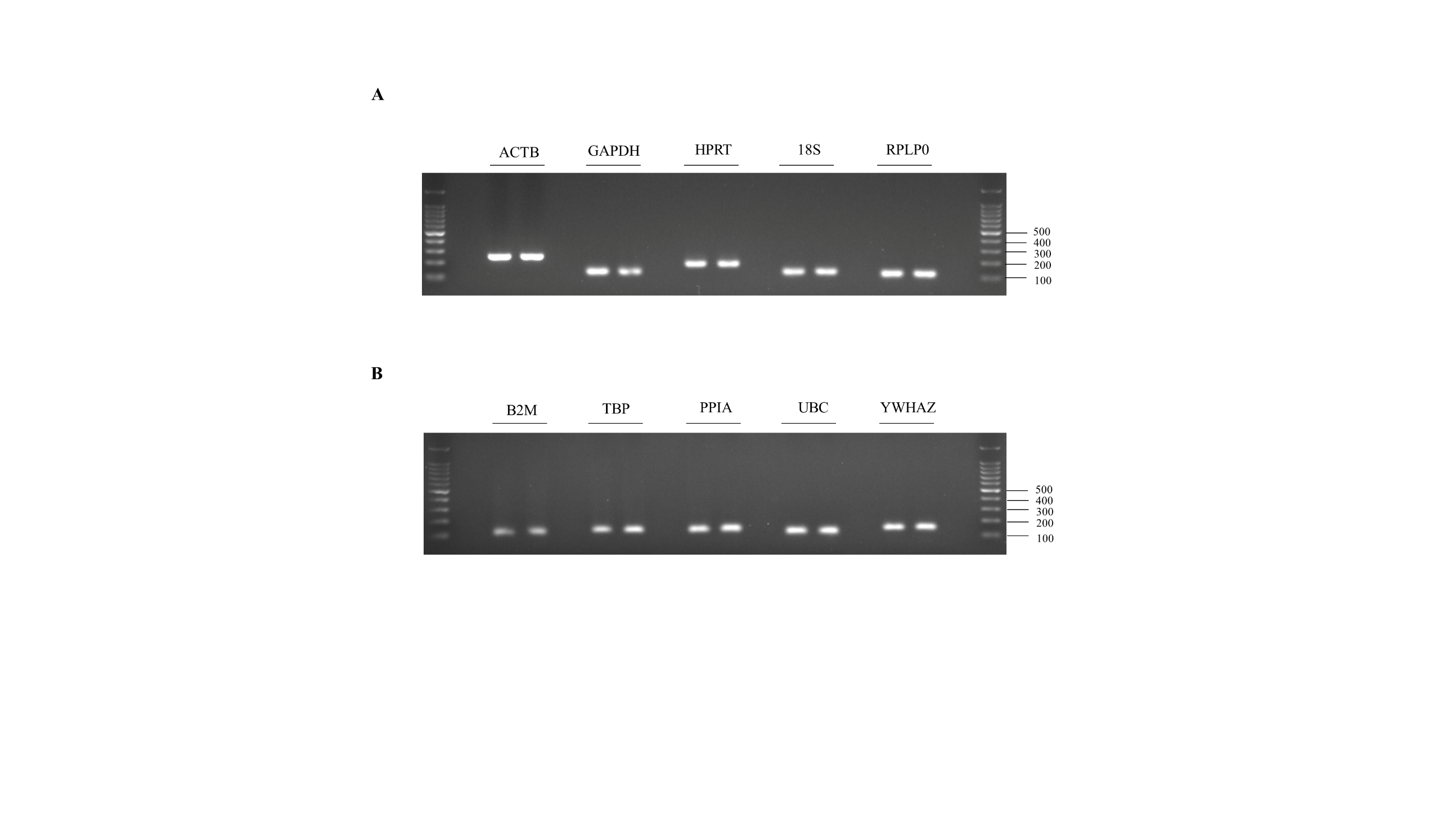
**

**Supplementary Figure 1. PCR product detection.** 100 bp DNA ladder are shown in both lanes to the far left and right, PCR products of ACTB, GAPDH, HPRT, 18S and RPLP0 (A), PCR products of B2M, TBP, PPIA, UBC and YWHAZ. Electrophoresis is in 2.0% agarose gel with TBE buffer at 100V for 0.5 hour at room temperature.

**
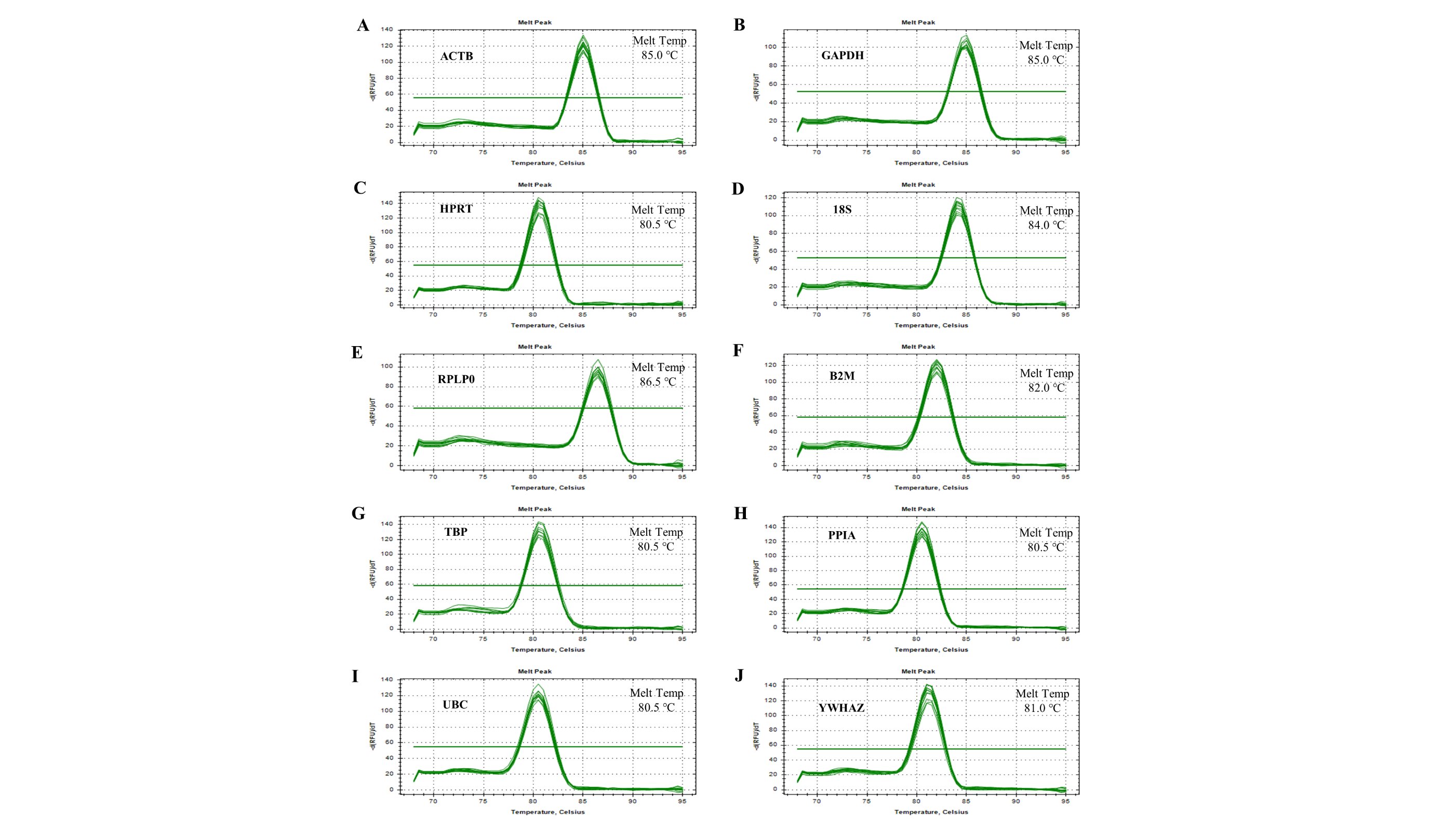
**

**Supplementary Figure 2. Melt curves for the qRT-PCR products.** (A) ACTB, (B) GAPDH, (C) HPRT, (D) 18S, (E) RPLP0, (F) B2M, (G)TBP, (H) PPIA, (I) UBC, (J) YWHAZ.

**
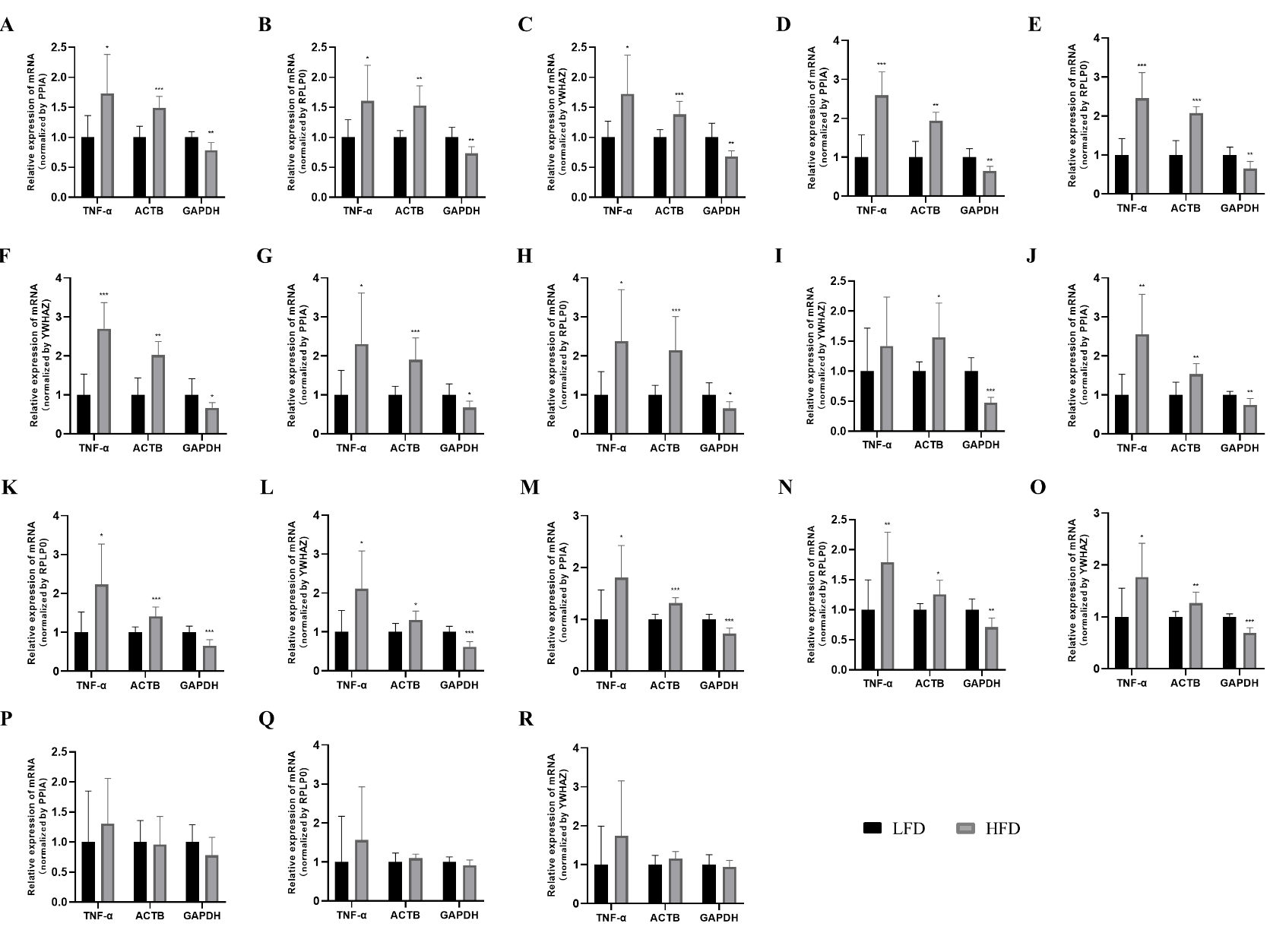
**

**Supplementary Figure 3. Changes in the mRNA expression of ACTB and GAPDH in high-fat-diet-induced obese mice.** Three to four-week-old C57BL/6J male mice were fed a high-fat diet (HFD) for 16 weeks, with a normal-fat diet (NFD) as a control. RT-qPCR was used to measure the mRNA levels of ACTB and GAPDH, with TNFα as a positive control, which was normalized to PPIA (A), RPLP0 (B) and YWHAZ (C) in the epididymal fat, PPIA (D), RPLP0 (E) and YWHAZ (F) in the perirenal fat, PPIA (G), RPLP0 (H) and YWHAZ (I) in the subcutaneous inguinal fat, PPIA (J), RPLP0 (K) and YWHAZ (L) in the subscapular brown adipose tissue, PPIA (M), RPLP0 (N) and YWHAZ (O) in the liver, and PPIA (P), RPLP0 (Q) and YWHAZ (R) in the femoral muscle. n=8 in each group. All data are presented as the means ± SD; * significantly different from the NFD group (p<0.05). **significantly different from the NFD group (p<0.01). *** significantly different from the NFD group (p<0.005).

## Supplementary Tables

**Supplementary Table 1. The diet composition for mice in this study.**

|  | **High fat diet (H10060)** | | **Normal fat diet (H10010)** | |
| --- | --- | --- | --- | --- |
| **Ingredients** | **g** | **kcal** | **g** | **kcal** |
| Casein | 189.6 | 758 | 189.6 | 758 |
| Cystine | 2.8 | 11 | 2.8 | 11 |
| Cornstarch | -- | -- | 298.6 | 1194 |
| Maltodextrin | 118.5 | 474 | 33.2 | 133 |
| Sucrose | 65.2 | 261 | 331.8 | 1327 |
| Fibrin | 47.4 | 0 | 47.4 | 0 |
| Soybean oil | 23.7 | 213 | 23.7 | 213 |
| Lard oil | 232.2 | 2090 | 19 | 171 |
| Mineral mixture (M1002) | 9.5 | 0 | 9.5 | 0 |
| Dicalcium phosphate | 12.3 | 0 | 12.3 | 0 |
| Calcium carbonate | 5.2 | 0 | 5.2 | 0 |
| Potassium citrate | 15.6 | 0 | 15.6 | 0 |
| Vitamin mixture (V1001) | 9.5 | 38 | 9.5 | 38 |
| Choline Bitartrate | 1.9 | 0 | 1.9 | 0 |
| Blue Dye | 0.05 | 0 | -- | -- |
| Yellow Dye | -- | -- | 0.05 | 0 |
| **Total** | **733.45** | **3845** | **1000.15** | **3845** |

**Supplementary Table 2. Quality control of RNA samples.**

| **Tissue**  **type** | **Time point** | **Concentration (μg/μl)** | **260nm/280nm** | **Tissue type** | **Time point** | **Concentration (μg/μl)** | **260nm/280nm** |
| --- | --- | --- | --- | --- | --- | --- | --- |
| epididymal fat | 4w-NFD | 0.30±0.08 | 1.95±0.02 | brown adipose tissue | 4w-NFD | 0.38±0.08 | 1.92±0.02 |
|  | 4w-HFD | 0.27±0.09 | 1.94±0.03 |  | 4w-HFD | 0.30±0.08 | 1.91±0.02 |
|  | 8w-NFD | 0.27±0.05 | 1.95±0.02 |  | 8w-NFD | 0.56±0.17 | 1.91±0.06 |
|  | 8w-HFD | 0.38±0.05 | 1.98±0.01 |  | 8w-HFD | 0.37±0.15 | 1.90±0.02 |
|  | 12w-NFD | 0.30±0.08 | 1.95±0.02 |  | 12w-NFD | 0.32±0.07 | 1.91±0.03 |
|  | 12w-HFD | 0.25±0.08 | 1.94±0.02 |  | 12w-HFD | 0.22±0.03 | 1.89±0.03 |
|  | 16w-NFD | 0.22±0.07 | 1.88±0.04 |  | 16w-NFD | 0.30±0.08 | 1.87±0.03 |
|  | 16w-HFD | 0.27±0.09 | 1.85±0.03 |  | 16w-HFD | 0.26±0.07 | 1.88±0.03 |
| perirenal fat | 4w-NFD | 0.54±0.16 | 2.02±0.02 | liver | 4w-NFD | 0.58±0.16 | 2.02±0.02 |
|  | 4w-HFD | 0.41±0.15 | 1.99±0.04 |  | 4w-HFD | 0.65±0.20 | 1.99±0.02 |
|  | 8w-NFD | 0.47±0.19 | 2.01±0.04 |  | 8w-NFD | 0.57±0.18 | 2.00±0.01 |
|  | 8w-HFD | 0.28±0.08 | 1.95±0.03 |  | 8w-HFD | 0.56±0.15 | 2.02±0.03 |
|  | 12w-NFD | 0.39±0.17 | 1.97±0.07 |  | 12w-NFD | 0.55±0.14 | 1.99±0.02 |
|  | 12w-HFD | 0.30±0.08 | 1.94±0.05 |  | 12w-HFD | 0.60±0.16 | 1.98±0.02 |
|  | 16w-NFD | 0.45±0.18 | 1.90±0.04 |  | 16w-NFD | 0.78±0.15 | 1.97±0.02 |
|  | 16w-HFD | 0.25±0.05 | 1.92±0.06 |  | 16w-HFD | 0.67±0.14 | 1.96±0.03 |
| subcutaneous inguinal fat | 4w-NFD | 0.67±0.13 | 1.97±0.02 | femoral muscle | 4w-NFD | 0.64±0.17 | 1.96±0.06 |
|  | 4w-HFD | 0.68±0.15 | 1.93±0.04 |  | 4w-HFD | 0.51±0.13 | 1.96±0.05 |
|  | 8w-NFD | 0.65±0.12 | 1.93±0.02 |  | 8w-NFD | 0.78±0.15 | 2.01±0.05 |
|  | 8w-HFD | 0.44±0.11 | 1.94±0.03 |  | 8w-HFD | 0.51±0.06 | 1.93±0.03 |
|  | 12w-NFD | 0.55±0.15 | 1.93±0.06 |  | 12w-NFD | 0.36±0.09 | 1.87±0.03 |
|  | 12w-HFD | 0.48±0.18 | 1.83±0.03 |  | 12w-HFD | 0.33±0.08 | 1.83±0.03 |
|  | 16w-NFD | 0.47±0.13 | 1.94±0.03 |  | 16w-NFD | 0.73±0.18 | 1.94±0.05 |
|  | 16w-HFD | 0.53±0.07 | 1.93±0.02 |  | 16w-HFD | 0.58±0.10 | 1.89±0.02 |

**Supplementary Table 3. Relative expression of each reference gene using the top four ranked reference genes for normalization in epididymal fat during the development of obesity.**

| **Time point** | **RG**  **TG** | RPLP0 | 18S | ACTB | B2M | GAPDH | TBP | HPRT | PPIA | UBC | YWHAZ |
| --- | --- | --- | --- | --- | --- | --- | --- | --- | --- | --- | --- |
| 4w | PPIA | 0.91 | 1.13 | 0.88 | 0.92 | 0.55** | 0.61** | 0.97 |  | 1.13 | 1.03 |
|  | YWHAZ | 0.84 | 1.08 | 0.82 | 0.85 | 0.52** | 0.57** | 0.94 | 0.93 | 1.05 |  |
|  | RPLP0 |  | 1.30 | 1.00 | 1.04 | 0.62** | 0.67** | 1.11 | 1.15 | 1.34 | 1.18 |
|  | 18S | 0.82 |  | 0.82 | 0.86 | 0.52** | 0.56** | 0.89 | 0.91 | 1.07 | 0.96 |
| 8w | RPLP0 |  | 1.57*** | 1.22* | 1.00 | 0.76* | 1.08 | 1.10 | 1.02 | 0.32*** | 1.17 |
|  | HPRT | 0.91 | 1.43*** | 1.11 | 0.91 | 0.68** | 0.98 |  | 0.93 | 0.29*** | 1.06 |
|  | B2M | 1.01 | 1.57*** | 1.22* |  | 0.76* | 1.08 | 1.10 | 1.02 | 0.32*** | 1.17 |
|  | PPIA | 1.00 | 1.56*** | 1.20* | 0.99 | 0.74* | 1.06 | 1.08 |  | 0.31*** | 1.12 |
| 12w | RPLP0 |  | 1.22* | 1.38** | 1.11 | 0.77** | 0.71*** | 1.19 | 1.01 | 1.16 | 0.91 |
|  | PPIA | 1.01 | 1.25 | 1.39* | 1.12 | 0.76* | 0.71** | 1.20* |  | 1.14 | 0.92 |
|  | B2M | 0.90 | 1.10 | 1.24* |  | 0.69*** | 0.64*** | 1.09 | 0.90 | 1.05 | 0.82* |
|  | YWHAZ | 1.07 | 1.30* | 1.47** | 1.19* | 0.81 | 0.77** | 1.30* | 1.08 | 1.21 |  |
| 16w | RPLP0 |  | 1.05 | 1.66** | 1.08 | 0.73** | 0.83* | 1.03 | 0.94 | 1.13 | 1.07 |
|  | PPIA | 1.08 | 1.13 | 1.71*** | 1.12 | 0.79** | 0.89 | 1.08 |  | 1.25 | 1.12 |
|  | HPRT | 1.01 | 1.05 | 1.58*** | 1.04 | 0.73** | 0.83 |  | 0.92 | 1.16 | 1.04 |
|  | YWHAZ | 0.95 | 0.99 | 1.53*** | 1.00 | 0.68** | 0.80* | 0.95 | 0.87 | 1.13 |  |

Note: Three to four-week-old C57BL/6J male mice were fed a high-fat diet (HFD), with a normal-fat diet (NFD) as a control. At 4, 8, 12, and 16 weeks after feeding, mice were sacrificed respectively, and organs and tissues were dissected. The mRNA expression of candidate reference genes were examined by RT-qPCR. The relative expression of other reference genes (TG, target genes) with the HFD feeding to the NFD feeding was determined with the top four reference genes (RG, reference genes) respectively as the invariant internal control. All data are presented as the means ± SD; n=8 in either the HFD or the NFD group at each time points. *compared with the NFD feeding group; p<0.05. **compared with the NFD feeding group, p<0.01; ***compared with the NFD feeding group, p<0.005.

**Supplementary Table 4. Relative expression of each reference gene using the top four ranked reference genes for normalization in perirenal fat during the development of obesity.**

| **Time point** | **RG**  **TG** | RPLP0 | 18S | ACTB | B2M | GAPDH | TBP | HPRT | PPIA | UBC | YWHAZ |
| --- | --- | --- | --- | --- | --- | --- | --- | --- | --- | --- | --- |
| 4w | RPLP0 |  | 1.37* | 1.28* | 1.10 | 0.68 | 0.94 | 1.36** | 1.10 | 1.53 | 1.06 |
|  | PPIA | 0.90 | 1.23 | 1.15 | 1.01 | 0.63* | 0.85 | 1.26* |  | 1.42 | 0.96 |
|  | YWHAZ | 0.95 | 1.29* | 1.21* | 1.03 | 0.64* | 0.87* | 1.30** | 1.06 | 1.54 |  |
|  | ACTB | 0.78* | 1.08 |  | 0.87 | 0.52* | 0.72** | 1.08 | 0.86 | 1.21 | 0.82* |
| 8w | RPLP0 |  | 1.17 | 1.45* | 1.01 | 0.43*** | 0.71*** | 1.32* | 1.06 | 0.81 | 0.96 |
|  | PPIA | 0.93 | 1.12 | 1.36* | 0.97 | 0.41*** | 0.67** | 1.25* |  | 0.76 | 0.89 |
|  | YWHAZ | 1.04 | 1.23* | 1.49*** | 1.01 | 0.48*** | 0.74* | 1.35* | 1.11 | 0.80 |  |
|  | 18S | 0.86 |  | 1.26 | 0.87 | 0.39*** | 0.61*** | 1.15 | 0.94 | 0.66 | 0.83 |
| 12w | PPIA | 0.93 | 0.97 | 1.55** | 0.98 | 0.46** | 0.66** | 1.37*** |  | 0.76 | 1.02 |
|  | RPLP0 |  | 1.02 | 1.69*** | 1.05 | 0.49** | 0.71** | 1.44*** | 1.07 | 0.76 | 1.11 |
|  | B2M | 0.95 | 0.98 | 1.58*** |  | 0.47** | 0.68** | 1.39*** | 1.02 | 0.79 | 1.04 |
|  | TBP | 1.39** | 1.46* | 2.36*** | 1.45** | 0.71* |  | 2.00*** | 1.48** | 1.00 | 1.56*** |
| 16w | PPIA | 0.96 | 0.71 | 1.90*** | 1.01 | 0.55** | 0.58** | 1.37*** |  | 3.88* | 0.89 |
|  | RPLP0 |  | 0.76 | 1.92*** | 1.01 | 0.55* | 0.59** | 1.37* | 1.01 | 3.74* | 0.92 |
|  | YWHAZ | 1.09 | 0.78 | 2.08*** | 1.12 | 0.60* | 0.64*** | 1.52*** | 1.11 | 4.51* |  |
|  | B2M | 0.87 | 0.61 | 1.94*** |  | 0.52* | 0.53*** | 1.35*** | 0.94 | 4.06* | 0.83 |

Note: Three to four-week-old C57BL/6J male mice were fed a high-fat diet (HFD), with a normal-fat diet (NFD) as a control. At 4, 8, 12, and 16 weeks after feeding, mice were sacrificed respectively, and organs and tissues were dissected. The mRNA expression of candidate reference genes were examined by RT-qPCR. The relative expression of other reference genes (TG, target genes) with the HFD feeding to the NFD feeding was determined with the top four reference genes (RG, reference genes) respectively as the invariant internal control. All data are presented as the means ± SD; n=8 in either the HFD or the NFD group at each time points. *compared with the NFD feeding group; p<0.05. **compared with the NFD feeding group, p<0.01; ***compared with the NFD feeding group, p<0.005.

**Supplementary Table 5. Relative expression of each reference gene using the top four ranked reference genes for normalization in subcutaneous inguinal fat during the development of obesity.**

| **Time point** | **RG**  **TG** | RPLP0 | 18S | ACTB | B2M | GAPDH | TBP | HPRT | PPIA | UBC | YWHAZ |
| --- | --- | --- | --- | --- | --- | --- | --- | --- | --- | --- | --- |
| 4w | PPIA | 1.11 | 2.70*** | 1.34* | 0.88 | 0.51*** | 1.11 | 1.01 |  | 1.53* | 0.96 |
|  | TBP | 1.02 | 2.44** | 1.21 | 0.80* | 0.47*** |  | 0.90 | 0.91 | 1.35 | 0.89 |
|  | YWHAZ | 1.14* | 2.86** | 1.38* | 0.93 | 0.53*** | 1.17 | 1.07 | 1.04 | 1.61* |  |
|  | HPRT | 1.17 | 2.68*** | 1.40 | 0.89 | 0.52*** | 1.12 |  | 1.02 | 1.52* | 1.01 |
| 8w | PPIA | 1.26 | 0.84 | 1.55* | 0.84* | 0.65* | 1.05 | 1.15 |  | 1.30 | 1.27* |
|  | RPLP0 |  | 0.67* | 1.30* | 0.70* | 0.49* | 0.83* | 0.96 | 0.83 | 1.05 | 1.02 |
|  | TBP | 1.21* | 0.82 | 1.53* | 0.85 | 0.64* |  | 1.17 | 1.02 | 1.30 | 1.24 |
|  | YWHAZ | 0.98 | 0.67* | 1.28* | 0.67* | 0.48** | 0.82 | 0.93 | 0.81 | 1.01 |  |
| 12w | HPRT | 0.76 | 1.29 | 1.39 | 1.17 | 0.42* | 0.57 |  | 0.81 | 2.23 | 0.80 |
|  | PPIA | 0.82 | 1.36 | 1.60* | 1.29 | 0.49*** | 0.74 | 1.01 |  | 2.03* | 0.92 |
|  | RPLP0 |  | 1.55* | 1.83** | 1.62* | 0.60* | 0.80 | 1.27 | 1.16 | 2.59* | 1.00 |
|  | B2M | 0.70 | 1.18 | 1.29 |  | 0.36*** | 0.51* | 0.83 | 0.75 | 1.56 | 0.75 |
| 16w | PPIA | 0.91 | 0.58* | 1.90** | 1.48** | 0.56** | 0.86 | 1.35*** |  | 1.24 | 1.24 |
|  | RPLP0 |  | 0.64* | 2.14** | 1.62* | 0.62* | 0.93 | 1.48* | 1.07 | 1.33 | 1.37 |
|  | YWHAZ | 0.74** | 0.48** | 1.56* | 1.16 | 0.45*** | 0.69*** | 1.08 | 0.79 | 0.99 |  |
|  | HPRT | 0.68* | 0.44* | 1.38* | 1.08 | 0.42** | 0.64*** |  | 0.74** | 0.90 | 0.93 |

Note: Three to four-week-old C57BL/6J male mice were fed a high-fat diet (HFD), with a normal-fat diet (NFD) as a control. At 4, 8, 12, and 16 weeks after feeding, mice were sacrificed respectively, and organs and tissues were dissected. The mRNA expression of candidate reference genes were examined by RT-qPCR. The relative expression of other reference genes (TG, target genes) with the HFD feeding to the NFD feeding was determined with the top four reference genes (RG, reference genes) respectively as the invariant internal control. All data are presented as the means ± SD; n=8 in either the HFD or the NFD group at each time points. *compared with the NFD feeding group; p<0.05. **compared with the NFD feeding group, p<0.01; ***compared with the NFD feeding group, p<0.005.

**Supplementary Table 6. Relative expression of each reference gene using the top four ranked reference genes for normalization in brown adipose tissue during the development of obesity.**

| **Time point** | **RG**  **TG** | RPLP0 | 18S | ACTB | B2M | GAPDH | TBP | HPRT | PPIA | UBC | YWHAZ |
| --- | --- | --- | --- | --- | --- | --- | --- | --- | --- | --- | --- |
| 4w | PPIA | 0.94 | 1.12* | 1.12* | 1.06 | 0.62*** | 1.05 |  | 0.84 | 0.81 | 1.00 |
|  | TBP | 0.90 | 1.07 | 1.07 | 0.99 | 0.59** |  | 0.79 | 0.95 | 0.77* | 0.95 |
|  | RPLP0 |  | 1.19 | 1.19* | 1.11 | 0.65* | 1.11 | 0.86 | 1.06 | 0.83 | 1.06 |
|  | YWHAZ | 0.95 | 1.14 | 1.14 | 1.06 | 0.62** | 1.05 | 0.83 | 1.01 | 0.83 |  |
| 8w | PPIA | 1.01 | 1,14 | 1.30** | 1.09 | 0.72*** | 1.04 | 0.87 |  | 0.72 | 0.98 |
|  | YWHAZ | 1.05 | 1.13 | 1.35** | 1.12 | 0.74*** | 1.09 | 0.89 | 1.04 | 0.72 |  |
|  | RPLP0 |  | 1.17 | 1.29* | 1.10 | 0.72*** | 1.03 | 0.87 | 1.00 | 0.75 | 0.98 |
|  | HPRT | 1.16 | 1.34 | 1.48** | 1.28* | 0.82* | 1.19 |  | 1.14 | 0.83 | 1.10 |
| 12w | RPLP0 |  | 1.05 | 1.33*** | 1.09 | 0.67** | 1.15 | 0.91 | 1.04 | 0.72* | 1.00 |
|  | PPIA | 1.00 | 1.03 | 1.34* | 1.09 | 0.64*** | 1.11 | 0.88* |  | 0.72* | 1.00 |
|  | YWHAZ | 0.98 | 1.02 | 1.32*** | 1.08 | 0.64* | 1.11 | 0.89 | 1.01 | 0.73* |  |
|  | HPRT | 1.15 | 1.21 | 1.54** | 1.24 | 0.73** | 1.29* |  | 1.15 | 0.83 | 1.15 |
| 16w | RPLP0 |  | 0.96 | 1.46*** | 1.04 | 0.62*** | 1.23* | 0.93 | 0.95 | 1.02 | 1.07 |
|  | YWHAZ | 0.94 | 0.90 | 1.36** | 0.96 | 0.58*** | 1.16 | 0.86 | 0.89 | 0.98 |  |
|  | HPRT | 1.08 | 1.03 | 1.58*** | 1.13 | 0.67*** | 1.31* |  | 1.02 | 1.12 | 1.14 |
|  | PPIA | 1.04 | 1.01 | 1.49** | 1.04 | 0.65*** | 1.29* | 0.95 |  | 1.10 | 1.11 |

Note: Three to four-week-old C57BL/6J male mice were fed a high-fat diet (HFD), with a normal-fat diet (NFD) as a control. At 4, 8, 12, and 16 weeks after feeding, mice were sacrificed respectively, and organs and tissues were dissected. The mRNA expression of candidate reference genes were examined by RT-qPCR. The relative expression of other reference genes (TG, target genes) with the HFD feeding to the NFD feeding was determined with the top four reference genes (RG, reference genes) respectively as the invariant internal control. All data are presented as the means ± SD; n=8 in either the HFD or the NFD group at each time points. *compared with the NFD feeding group; p<0.05. **compared with the NFD feeding group, p<0.01; ***compared with the NFD feeding group, p<0.005.

**Supplementary Table 7. Relative expression of each reference gene using the top four ranked reference genes for normalization in liver during the development of obesity.**

| **Time point** | **RG**  **TG** | RPLP0 | 18S | ACTB | B2M | GAPDH | TBP | HPRT | PPIA | UBC | YWHAZ |
| --- | --- | --- | --- | --- | --- | --- | --- | --- | --- | --- | --- |
| 4w | B2M | 1.00 | 1.44 | 1.01 |  | 0.78* | 0.96 | 1.17 | 0.94 | 1.27 | 1.04 |
|  | RPLP0 |  | 1.44 | 1.00 | 1.00 | 0.78* | 0.96 | 1.16 | 0.94 | 1.24 | 1.04 |
|  | PPIA | 1.06 | 1.55 | 1.06 | 1.05 | 0.83** | 1.00 | 1.24* |  | 1.35 | 1.10 |
|  | ACTB | 1.00 | 1.48 |  | 1.01 | 0.78* | 0.95 | 1.17 | 0.94 | 1.28 | 1.05 |
| 8w | HPRT | 0.94 | 1.74*** | 1.02 | 1.13 | 0.81* | 1.24 |  | 0.98 | 0.36* | 0.94 |
|  | PPIA | 0.96 | 1.75*** | 1.04 | 1.15 | 0.83* | 1.25 | 1.02 |  | 0.36* | 0.95 |
|  | YWHAZ | 1.01 | 1.90*** | 1.10 | 1.21* | 0.88 | 1.32** | 1.07 | 1.05 | 0.37* |  |
|  | RPLP0 |  | 1.83*** | 1.08 | 1.20* | 0.87* | 1.33 | 1.07 | 1.05 | 0.41* | 1.00 |
| 12w | PPIA | 0.97 | 1.93*** | 1.04 | 0.96 | 0.72*** | 1.07 | 1.13 |  | 0.53* | 1.10 |
|  | RPLP0 |  | 2.01*** | 1.05 | 0.99 | 0.74** | 1.11 | 1.17 | 1.03 | 0.54* | 1.14 |
|  | HPRT | 0.86 | 1.68*** | 0.92 | 0.85* | 0.64*** | 0.95 |  | 0.89* | 0.47** | 0.97 |
|  | TBP | 0.91 | 1.79*** | 0.99 | 0.90 | 0.67*** |  | 1.06 | 0.94 | 0.49* | 1.04 |
| 16w | YWHAZ | 1.02 | 1.23* | 1.26* | 1.02 | 0.66*** | 1.16* | 1.07 | 0.99 | 0.48** | 0.98 |
|  | RPLP0 |  | 1.23* | 1.25* | 1.02 | 0.66** | 1.15 | 1.07 | 0.97 | 0.46** | 1.00 |
|  | PPIA | 1.03 | 1.24* | 1.27** | 1.04 | 0.67*** | 1.18* | 1.10 |  | 0.47** | 1.02 |
|  | HPRT | 0.97 | 1.16 | 1.20 | 0.95 | 0.63*** | 1.10 |  | 0.94 | 0.47* | 0.94 |

Note: Three to four-week-old C57BL/6J male mice were fed a high-fat diet (HFD), with a normal-fat diet (NFD) as a control. At 4, 8, 12, and 16 weeks after feeding, mice were sacrificed respectively, and organs and tissues were dissected. The mRNA expression of candidate reference genes were examined by RT-qPCR. The relative expression of other reference genes (TG, target genes) with the HFD feeding to the NFD feeding was determined with the top four reference genes (RG, reference genes) respectively as the invariant internal control. All data are presented as the means ± SD; n=8 in either the HFD or the NFD group at each time points. *compared with the NFD feeding group; p<0.05. **compared with the NFD feeding group, p<0.01; ***compared with the NFD feeding group, p<0.005.

**Supplementary Table 8. Relative expression of each reference gene using the top four ranked reference genes for normalization in femoral muscle during the development of obesity.**

| **Time point** | **RG**  **TG** | RPLP0 | 18S | ACTB | B2M | GAPDH | TBP | HPRT | PPIA | UBC | YWHAZ |
| --- | --- | --- | --- | --- | --- | --- | --- | --- | --- | --- | --- |
| 4w | RPLP0 |  | 1.30* | 1.26* | 0.92 | 1.04 | 1.02 | 1.01 | 0.95 | 0.64 | 1.13 |
|  | HPRT | 0.98 | 1.27 | 1.24* | 0.95 | 1.02 | 1.01 |  | 0.96 | 0.64 | 1.12 |
|  | PPIA | 0.99 | 1.20* | 1.27 | 1.01 | 1.03 | 1.03 | 1.03 |  | 0.71 | 1.14 |
|  | TBP | 0.98 | 1.24 | 1.23* | 0.91 | 1.03 |  | 1.00 | 0.94 | 0.63 | 1.11 |
| 8w | HPRT | 1.20 | 1.71** | 0.95 | 1.34* | 1.01 | 0.92 |  | 1.08 | 0.30*** | 1.10 |
|  | PPIA | 1.08 | 1.49* | 0.88 | 1.27* | 0.97 | 0.87 | 0.96 |  | 0.28*** | 1.00 |
|  | YWHAZ | 1.06 | 1.48** | 0.90 | 1.29* | 0.98 | 0.87 | 0.96 | 0.99 | 0.28*** |  |
|  | GAPDH | 1.14 | 1.63** | 0.93 | 1.31** |  | 0.89 | 0.97 | 1.04 | 0.29*** | 1.07 |
| 12w | YWHAZ | 1.15 | 1.08 | 1.02 | 0.84 | 0.92 | 0.90 | 0.83 | 0.95 | 0.17* |  |
|  | TBP | 1.31 | 1.22* | 1.16 | 0.97 | 1.05 |  | 0.95 | 1.08 | 0.20* | 1.16 |
|  | PPIA | 1.23* | 1.16 | 1.08 | 0.92 | 0.99 | 0.95 | 0.89 |  | 0.19* | 1.10 |
|  | HPRT | 1.33* | 1.25 | 1.17 | 1.02 | 1.08 | 1.05 |  | 1.11 | 0.23* | 1.19 |
| 16w | RPLP0 |  | 1.47*** | 1.10 | 1.14 | 0.96 | 0.84 | 0.73* | 1.28 | 2.03 | 0.94 |
|  | YWHAZ | 1.04 | 1.52** | 1.16 | 1.21* | 0.99 | 0.88 | 0.77* | 1.34 | 1.99* |  |
|  | B2M | 0.85 | 1.25 | 0.96 |  | 0.81 | 0.71 | 0.62* | 1.11 | 1.57 | 0.82* |
|  | PPIA | 0.87 | 1.26 | 0.96 | 1.00 | 0.81 | 0.72* | 0.63* |  | 1.60 | 0.82 |

Note: Three to four-week-old C57BL/6J male mice were fed a high-fat diet (HFD), with a normal-fat diet (NFD) as a control. At 4, 8, 12, and 16 weeks after feeding, mice were sacrificed respectively, and organs and tissues were dissected. The mRNA expression of candidate reference genes were examined by RT-qPCR. The relative expression of other reference genes (TG, target genes) with the HFD feeding to the NFD feeding was determined with the top four reference genes (RG, reference genes) respectively as the invariant internal control. All data are presented as the means ± SD; n=8 in either the HFD or the NFD group at each time points. *compared with the NFD feeding group; p<0.05. **compared with the NFD feeding group, p<0.01; ***compared with the NFD feeding group, p<0.005.
